# Regulation of Pfh1 helicase activity by nucleic acid interactions and mitochondrial SSB

**DOI:** 10.1073/pnas.2602528123

**Published:** 2026-05-18

**Authors:** María Ortiz-Rodríguez, Saurabh P. Singh, Francisco J. Cao-García, Roberto Galletto, Borja Ibarra

**Affiliations:** ^a^Instituto Madrileño de Estudios Avanzados en Nanociencia, Madrid 28049, Spain; ^b^Department of Biochemistry and Molecular Biophysics, Washington University School of Medicine, St. Louis, MO 63110; ^c^ Departamento de Estructura de la Materia, Física Térmica y Electrónica, Universidad Complutense de Madrid, Madrid 28040, Spain; ^d^Nanobiotecnología (IMDEA-Nanociencia), Unidad Asociada al Centro Nacional de Biotecnología, Madrid 28049, Spain

**Keywords:** Pfh1 helicase, DNA unwinding kinetics, single-molecule manipulation, ATP-dependent helicase activity, mitochondrial SSB interactions

## Abstract

Pif1-family helicases are essential for proper nuclear and mitochondrial genome maintenance, yet the regulation of their activities remains incompletely understood. Here, we use single-molecule manipulation and visualization techniques to dissect the real-time mechanochemical behavior of Pfh1, the sole Pif1-family helicase in *Schizosaccharomyces pombe.* We systematically varied force, ATP concentration, fork composition, and the single-stranded DNA–binding protein spRim1, to quantify the unwinding and single-stranded DNA translocation properties of Pfh1. We find that Pfh1 operates through unwinding-rewinding cycles during which coordinated interactions with both DNA strands at the fork optimize ATP utilization. Contacts with the translocating strand modulate ATP affinity, while interactions with the displaced strand control maximum unwinding velocity. Binding of spRim1 to the displaced strand disrupts the latter interactions, increasing the unwinding velocity. Stable interactions of the helicase with both strands at the fork may limit unwinding processivity to ~20 bp, eventually triggering transition to rewinding. Rewinding proceeds through an ATP-dependent process that is incompatible with strand switching, in which ATP turnover modulates DNA contacts and rewinding rate. Binding of spRim1 to the displaced strand further accelerates rewinding, possibly by competing with helicase–DNA interactions, and facilitates recovery of the active unwinding conformation once the fork has rewound. Together, these findings suggest that Pfh1 balances unwinding and rewinding through coordinated ATP-dependent strand interactions, providing insight into how Pif1-family helicases are controlled at replication forks.

## Significance

Pif1-family helicases play key roles in genome maintenance, yet how their activities are regulated at replication forks remains poorly understood. Using single-molecule approaches, we show that the fission yeast helicase Pfh1 operates through force- and ATP-sensitive unwinding–rewinding cycles that differ from those reported for its budding-yeast homolog Pif1. During unwinding, stable interactions with both DNA strands at the replication fork enhance catalytic efficiency while imposing an intrinsic limit to processivity, revealing an active regulatory role for the displaced strand. During rewinding, Pfh1 follows an ATP-dependent sliding-back mechanism that maintains helicase association with the fork. Together, these findings provide a mechanistic framework explaining how Pfh1 promotes replication fork progression without disrupting replisome organization.

The Pif1 family of helicases is comprised of 5′-3′ directed, ATP-dependent, superfamily (SF) 1B DNA helicases that are evolutionarily conserved from bacteria to humans (1, 2). Members of this family are multifunctional helicases essential for both nuclear and mitochondrial DNA maintenance. Their roles include inhibition of telomerase activity (3, 4), processing of Okazaki fragments (5–8), promotion of break-induced replication (9, 10), prevention of replication pausing at G-quadruplex (11–15), and maintenance of mitochondrial DNA integrity (16–19).

The fission yeast *Schizosaccharomyces pombe*, like humans and most metazoans, encodes a single Pif1 family helicase, Pfh1 (1, 17, 20, 21). In contrast, *Saccharomyces cerevisiae* possesses two paralogs, Pif1 and Rrm3, which exhibit distinct roles in modulating replication intermediates (13, 22–25). Although *S. cerevisiae* cells lacking both Pif1 and Rrm3 remain viable (22), Pfh1 is essential in *S. pombe* (16, 17, 20, 26–28). Because Pfh1 must carry out the combined roles of Pif1 and Rrm3, a key open question is which biochemical and mechanistic features set Pfh1 apart from its budding-yeast counterparts and allow it to perform these functions on its own.

Ensemble experiments indicate that Pfh1 exhibits limited intrinsic DNA unwinding activity in the absence of a trap to prevent reannealing of the unwound strands, a behavior similar to that reported for Pif1 (29, 30). This observation is consistent with a rewinding activity counteracting unwinding. Consistent with this interpretation, single-molecule FRET studies have shown that Pfh1, like Pif1, unwinds DNA through repetitive cycles of partial unwinding followed by rewinding (31). Such repetitive unwinding-rewinding dynamics appear to be conserved across many SF1 and SF2 helicase members (32). For Pif1 family helicases, because of their 5′-3′ directionality, these cycles may serve regulatory roles, enabling substantial unwinding only when DNA synthesis occurs concomitantly on the opposite strand. This mechanism would effectively couple DNA unwinding to DNA synthesis, facilitating transient disruption of stable G-quadruplex structures during replication (33, 34) or enabling long-range DNA synthesis during break-induced replication (10, 35, 36).

Notably, single-molecule manipulation studies have revealed that the unwinding/rewinding kinetics of SF1/SF2 helicases responded differently to external force when it is applied to destabilize the DNA fork. For example, both the unwinding velocity and processivity of Pif1 increase with force (37), whereas force has no effect on some helicases [i.e., Rec Q, NS3, (38, 39)] or is even detrimental for others [i.e., UvrD, (40)]. In addition, the mechanism underlying rewinding is also not fully understood. A strand-switching model, whereby the helicase transitions to the displaced strand and translocates away from the fork, has been proposed for several SF1/SF2 helicases, including Pif1 (37, 41, 42). However, our recent single-molecule FRET studies argued against strand-switching as the dominant mechanism underlying Pif1- and Pfh1-depedent rewinding on DNA fork-like substrates (31). Together, these differences suggest fundamental mechanistic differences between the DNA unwinding and rewinding mechanisms of helicases even within the same family.

In this study, we combine single-molecule manipulation and visualization approaches to dissect key mechanistic aspects of Pfh1 activity, including the determinants of unwinding processivity, the nature of the rewinding events, and how these processes are regulated by ATP turnover, helicase–DNA interactions and protein cofactors. Among the latter, of particular interest is the role of single-stranded DNA-binding (SSB) proteins, which modulate the activities of several helicases (43–48). For instance, Pif1 interacts with and is stimulated by the S. *cerevisiae* mitochondrial SSB Rim1 in vitro (49), suggesting coordinated roles in mitochondrial DNA metabolism. However, it remains unknown whether Pfh1 is similarly stimulated by its mitochondrial SSB counterpart (spRim1), and if so, how such stimulation influences Pfh1 unwinding and rewinding kinetics.

## Results

### Detection of Pfh1 Activities at the Single-Molecule Level.

**Fig. 1.**
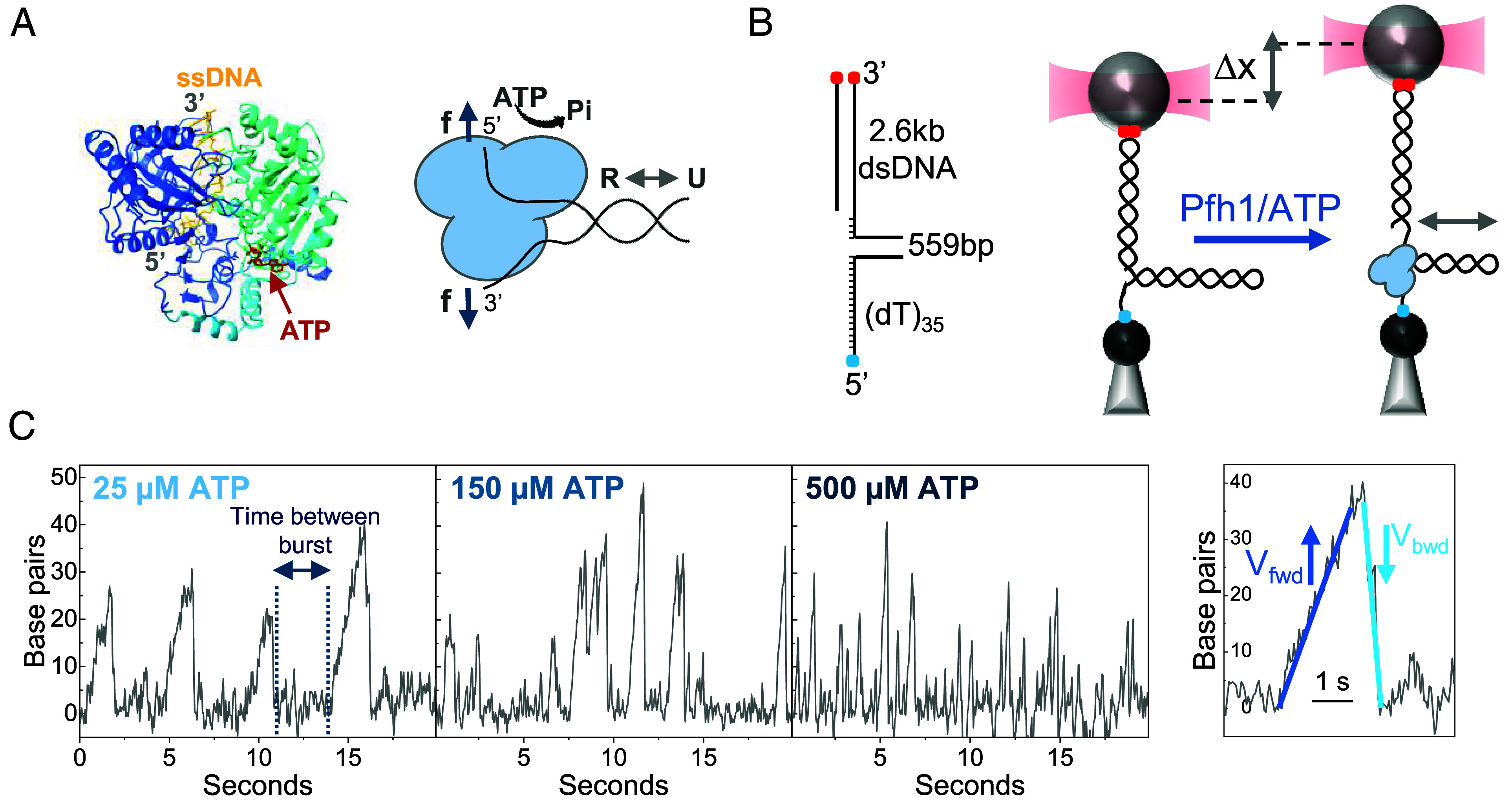
Experimental setup. (*A*, *Left*) Pfh1 central helicase domain structure bound to ssDNA and ATP was prepared by superimposing AlphaFold2 (58) Pfh1 prediction with the human Pif1 helicase bound to AMP-PNP (PDB 6HPH). (*Right*) Representation of Pfh1 at a fork junction, where it undergoes unwinding (U)-rewinding (R) cycles with 5′-3′ polarity in the presence of ATP. Blue arrows indicate the direction of application of external force (f). (*B*, *Left*) The fork-like DNA construct consists of a 559 bp DNA hairpin (*SI Appendix*, Table S2 for sequence) with a ∼2.6-kb long dsDNA handle labeled with digoxigenin (red dots) and a 5′-(dT)_35_ labeled with biotin (blue dot). The single-stranded (dT)_35_ tail serves as a helicase loading site. (*Right*) Schematic of the optical trapping assay. A single DNA construct is tethered between two functionalized microspheres. In the presence of Pfh1 (blue) and ATP, helicase activity was monitored as a change in tether extension (Δx) under constant force. The bidirectional gray arrow indicates Pfh1-mediated unwinding/rewinding cycles at the fork. (*C*, *Left*) Representative traces showing repetitive unwinding–rewinding cycles at several ATP concentrations with little net baseline change (force, *f* = 8 pN). (*Right*) Zoom-in unwinding-rewinding event (25 µM ATP, 8 pN) showing that forward unwinding (*V_fwd_*) and backward rewinding (*V_bwd_*) velocities were calculated from the forward (dark blue) and backward (light blue) segments within each burst (*Materials and Methods* and *SI Appendix*, Fig. S4).

Base-pair stability and protein–DNA fork interactions are key determinants of how helicases couple ATP turnover to DNA unwinding (50–56). To investigate how these factors influence the activity of the *S. pombe* Pfh1 helicase, we used dual-beam counterpropagating optical tweezers (57) to monitor, in real-time, the unwinding kinetics of Pfh1 (2 or 20 nM) on individual DNA hairpins under varying ATP concentrations (5 to 1,000 µM) (Fig. 1 *A* and *B* and *Materials and Methods*). Because force applied to the complementary strands of the DNA fork modulates both base-pair stability and protein–DNA interactions (37, 50–54), we performed the experiments at several constant forces (5 ≤ *f* < 11 pN), a range that maintains the hairpin in the closed state while allowing us to probe force-dependent effects.

The real-time kinetics of the helicase were followed by monitoring changes in the end-to-end distance of a DNA hairpin tethered between two beads in the optical tweezers (Fig. 1*B* and *Materials and Methods*). Pfh1 activity traces typically showed repetitive bursts of DNA extension followed by rapid compaction back to the original length, which we attribute to DNA unwinding-rewinding cycles (Fig. 1*C*). Unwinding–rewinding bursts persisted for several minutes after washing the chamber with a helicase-free buffer, demonstrating that the cycles arise from the activity of a single Pfh1 molecule (*SI Appendix*, Fig. S1).This behavior is consistent with unwinding-rewinding cycles reported for this helicase using smFRET in the absence of force (31). Analysis of unwinding and rewinding events is presented separately below.

### ATP Concentration and Force Dependencies of Pfh1’s DNA Unwinding Kinetics.

**Fig. 2.**
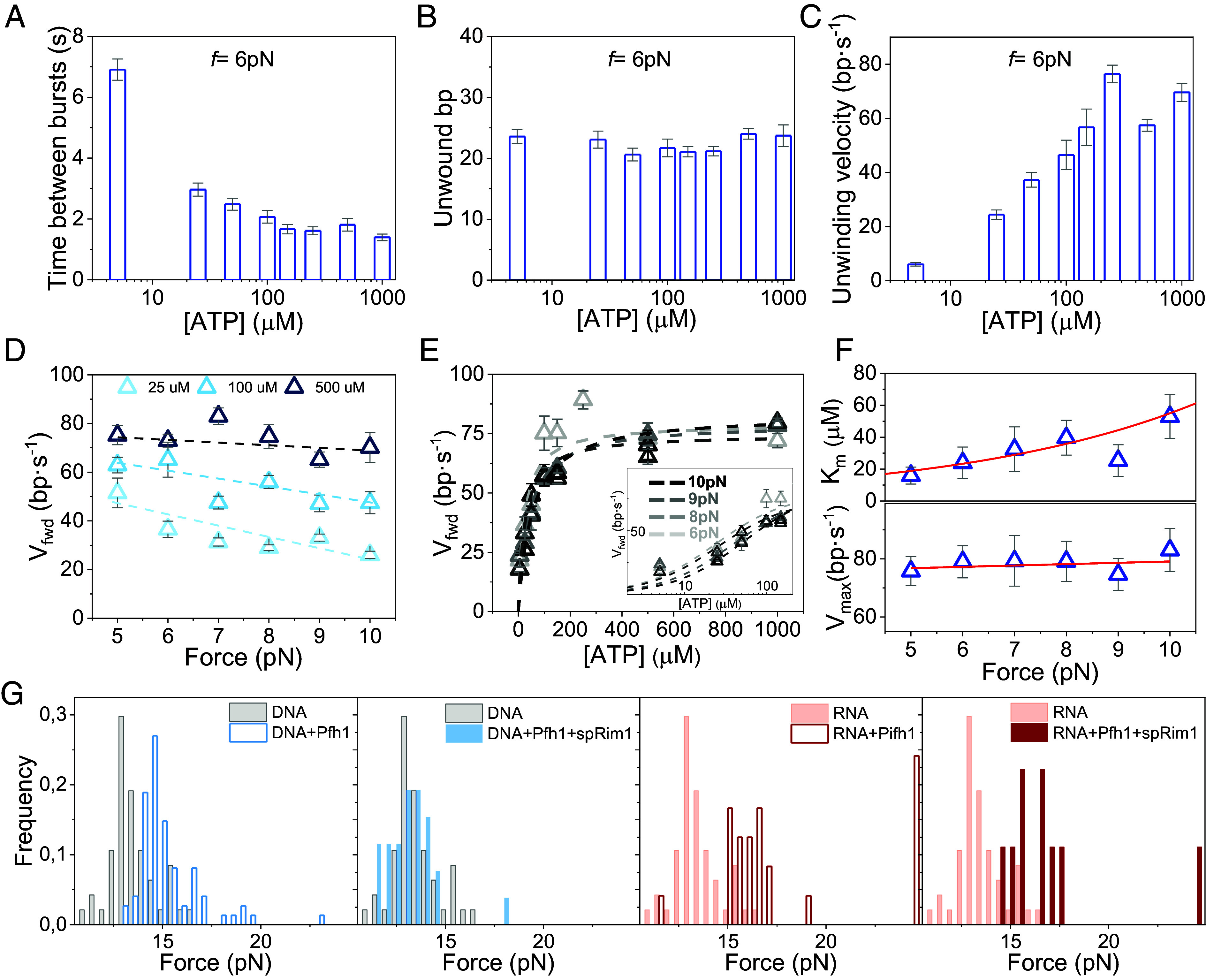
ATP concentration and force dependencies of Pfh1 unwinding activity. (*A*) Average time between consecutive unwinding events as a function of ATP concentration (*f* = 6 pN, *N* = 705). Data across all forces and ATP concentrations are shown in *SI Appendix*, Fig. S1. (*B* and *C*) ATP concentration dependencies of the average number of unwound base pairs (*B*) and average unwinding rates (*C*) at *f* = 6 pN (*N* = 705). Data across all forces and ATP concentrations are shown in *SI Appendix*, Fig. S2. (*D*) Forward unwinding velocity (*V_fwd_*) as a function of force at different ATP concentrations. Dashed lines are shown as a guide to the eye and do not represent fits. (*E*) Forward unwinding velocity (*V_fwd_*) as a function of ATP concentration at different applied forces (gray scale). *Inset*: zoom of the data at subsaturating ATP concentrations. Data were fitted with the Michaelis–Menten equation (dash lines). (*F*) Force dependencies of Michaelis–Menten parameters *K_m_*(*f*) and *V_max._*In the *Top* panel, the red line is the fit to the data with Eq. **1** (*Materials and Methods*). Best fit indicate a conformational change of ~0.8 nm along the pulling coordinate. In the *Bottom* panel, the red line panel represents the linear fit to the data (slope, *m* = 0.5 ± 0.7; *V_max_* = 75 ± 5 bp/s). (*G*) Unzipping force distributions of the DNA and RNA–DNA hairpins in the absence and presence of Pfh1 or Pfh1 + spRim1.

Analysis of the unwinding bursts (total number of bursts analyzed, *N* = 5,190, *SI Appendix*, Table S1) revealed that the average time intervals between consecutive unwinding bursts were unaffected by force or helicase concentration (*SI Appendix*, Fig. S2) but shortened with increasing ATP concentrations (Figs. 1*C* and 2*A*). This pattern is consistent with the observation that a single Pfh1 helicase repeatedly unwinds and rewinds DNA in an ATP-driven cycle, regardless of applied force.

Analysis of the amplitude of the unwinding bursts showed that Pfh1 unwinds an average of 22 ± 0.5 base pairs (bp) before rewinding to the starting position, regardless of protein concentration, the applied force, and ATP concentration (Fig. 2*B* and *SI Appendix*, Fig. S2). The total unwinding length (processivity) was substantially shorter than the full hairpin substrate (559 bp) with unwinding typically terminating in the initial region of the DNA that is free of GC clusters (*Materials and Methods* and *SI Appendix*, Table S2). This short processivity is consistent with previous observations for both Pfh1 and Pif1 in the absence of force using smFRET (31, 37).

As expected for a motor protein-driven process, the average velocity was independent on protein concentration (*SI Appendix*, Fig. S3) but depended on ATP concentration at all forces, increasing from ~10 to ~70 bp·s^−1^ as the ATP concentration increases from 5 to 1,000 µM (Fig. 2*C* and *SI Appendix*, Fig. S3). The fact that unwinding velocity depends on ATP concentration, while processivity does not, indicates that these two processes are uncoupled and that processivity is not governed by a time-dependent stochastic process.

Interestingly, both the average unwinding velocity and the forward unwinding velocity (*V_fwd_*), defined as the average velocity calculated after excluding transient pauses and brief backward events from each unwinding burst (*Materials and Methods*, Fig. 1*C* and *SI Appendix*, Fig. S4), decreased gradually with increasing force at low ATP concentrations (<150 µM). In contrast, no significant force dependence was observed at high ATP concentrations (≥500 µM) (Fig. 2*D* and *SI Appendix*, Fig. S3). These results suggest that applied force modulates the apparent ATP affinity of the helicase.

In fact, fit of *V_fwd_* as a function of ATP concentration at different forces using the Michaelis–Menten equation (Fig. 2*E*) yielded a force-dependent apparent Michaelis constant, *K_m_*(*f*), and a maximal unwinding velocity that was mainly force independent, *V_max_* = 75 ± 5 bp·s^−1^ (Fig. 2*F*). For a helicase that exhibits a kinetic step size of 1 bp, at the single-molecule level *V_max_* can be approximated as the maximal unwinding rate, and it is therefore proportional to *k_cat_*. Overall, these results indicate that force applied to the DNA decreases the catalytic efficiency (*V_max_*/*K*_m_(*f*), *SI Appendix*, Fig. S5) or the helicase’s capacity to couple ATP binding/hydrolysis energy to DNA unwinding, by decreasing its affinity for ATP.

An Arrhenius-type force dependence of *K_m_*(*f*), corresponding to a conformational change of *d* = 0.8 ± 0.3 nm [*Materials and Methods*, (59)] accurately describes the increase of *K_m_*(*f*) with force (Fig. 2*E*), and the concomitant decrease in catalytic efficiency (*SI Appendix*, Fig. S5). This characteristic distance *d* suggests that force induces the release of at least two ssDNA nucleotides (∼0.34 nm/nt) that initially interact with the helicase, thereby reducing ATP affinity and catalytic efficiency. Extrapolation of the fits to zero force yields an apparent *K_m_* of 6.5 ± 4.5 µM and a catalytic efficiency of 12.5 ± 2 µM^−1^ s^−1^ in the absence of force under our experimental conditions (*SI Appendix*, Fig. S5).

Among other interpretations, the observed force independence of the unwinding velocity at saturating ATP concentrations may suggest that Pfh1 shields the DNA duplex from the applied force, which would otherwise reduce base-pair stability and thereby facilitate helicase advance. To test this hypothesis, we measured the force required to unzip the hairpin after the helicase had initiated unwinding in the presence of ATP (Fig. 2*G* and *SI Appendix*, Fig. S6). Under these conditions, ~31% of hairpins unzip at forces more than two standard deviations above the mean unzipping force measured in the absence of helicase (Fig. 2*G*). This shift toward higher unzipping forces indicates that during unwinding Pfh1 reduces the effective destabilizing influence of the applied force, probably by stabilizing contacts with both DNA strands. Such strand engagement would explain the observed force independence of the unwinding velocity.

### spRim1 Promotes the DNA Unwinding Kinetics of Pfh1.

Next, we measured the effect of the *S. pombe* mitochondrial single-stranded DNA binding protein spRim1 (2 nM), on the real-time DNA unwinding kinetics of Pfh1 (2 nM), under varying force and ATP concentrations (total number of unwinding bursts analyzed, *N* = 7,462, *SI Appendix*, Table S1).

**Fig. 3.**
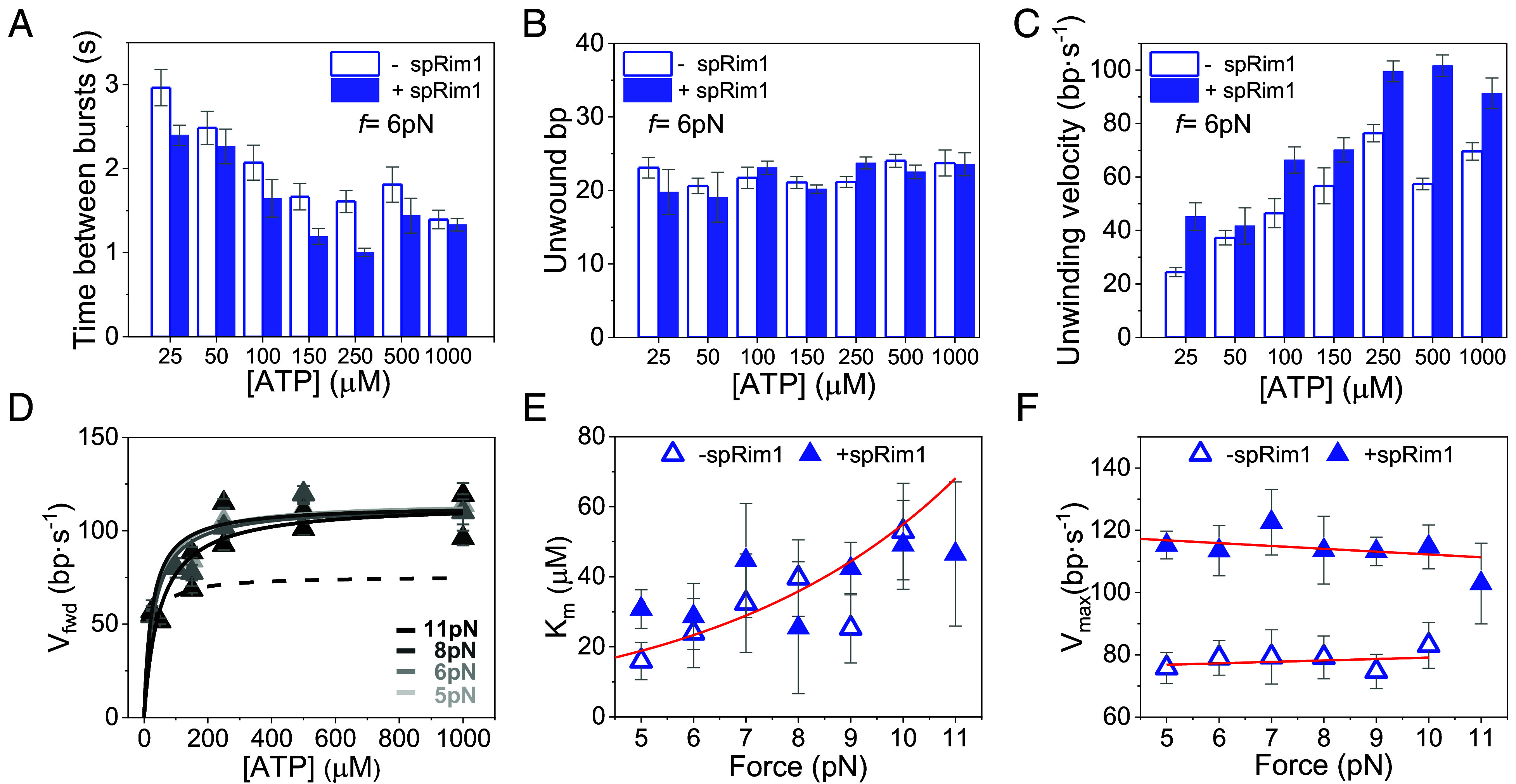
Effect of spRim1 on the DNA unwinding kinetics of Pfh1. (*A*) Average time between consecutive unwinding events in the absence (empty columns) and presence (filled columns) of spRim1 (2 nM) as a function of ATP concentration (*f* = 6 pN, *N* = 1,091). (*B* and *C*) ATP concentration dependencies of the average number of unwound base pairs (*B*) and average unwinding velocity (*C*) at *f* = 6 pN (*N* = 1,091). Data across all forces and ATP concentrations are shown in *SI Appendix*, Fig. S7. (*D*) Forward unwinding velocity (*V_fwd_*) in the presence of spRim1 as a function of ATP concentration at different applied forces (gray scale). Data were fitted with the Michaelis–Menten equation (solid lines). For comparison, dashed line shows the Michaelis–Menten fit to data in the absence of spRim1 (*f* = 9 pN). (*E* and *F*) Force dependencies of Michaelis–Menten parameters *K_m_* (*E*) and *V_max_* (*F*) in the absence (empty symbols) and presence of spRim1 (solid symbols). Red line in *E* shows the fit of Eq. **1** (*Materials and Methods*) to data in the absence of the SSB. Red lines in *F* represent linear fits to the data (without spRim1, *m* = 0.5 ± 0.7, *V_max_* = 75 ± 5 bp/s; with spRim1, m = −0.9 ± 0.7, *V_max_* = 115 ± 2 bp/s). For all data plots error bars show SEM.

The presence of spRim1 decreased the average time interval between bursts across all ATP concentrations, independent of force (Fig. 3*A* and *SI Appendix*, Fig. S2). This observation highlights the role of spRim1 in facilitating the recovery of Pfh1 unwinding activity following rewinding.

Analysis of individual unwinding traces revealed that spRim1 did not affect the unwinding processivity of Pfh1, which remained unchanged at 21 ± 0.5 bp and independent of both force and ATP concentration, as measured in the absence of the SSB (Fig. 3*B* and *SI Appendix*, Fig. S7). However, spRim1 increased significantly the unwinding velocity across all forces and ATP concentrations, Fig. 3 *C* and *D*. As observed in the absence of the SSB, the average velocity remained force-independent at saturating ATP concentrations (*SI Appendix*, Fig. S7) and increased with ATP concentration (Fig. 3*C*).

Fitting the forward unwinding velocity in the presence of spRim1 to the Michaelis–Menten equation (Fig. 3*D*) revealed that the SSB did not significantly alter the force-dependent *K_m_*(*f*) (Fig. 3*E*) but increased *V_max_* by ~1.5-fold (from 75 ± 5 bp/s without SSB to 115 ± 2 bp/s with SSB, Fig. 3*F*). This suggests that the observed increase in *V_max_* results from a mechanism other than modulation of the affinity of the helicase for ATP (*Discussion*). Moreover, human mtSSB stimulated the unwinding velocity of Pfh1 to a similar extent as spRim1 (*SI Appendix*, Fig. S7), indicating that stimulation is mediated by SSB binding to DNA rather than species-specific protein–protein interactions.

Finally, we note that spRim1 restored the unzipping force of the hairpin close to the value in the absence of the helicase, strongly suggesting that spRim1 destabilizes the interactions of Pfh1 with the fork (Fig. 2*G*).

### Pfh1 Exhibits Greater Catalytic Efficiency in DNA Unwinding than in ssDNA Translocation.

**Fig. 4.**
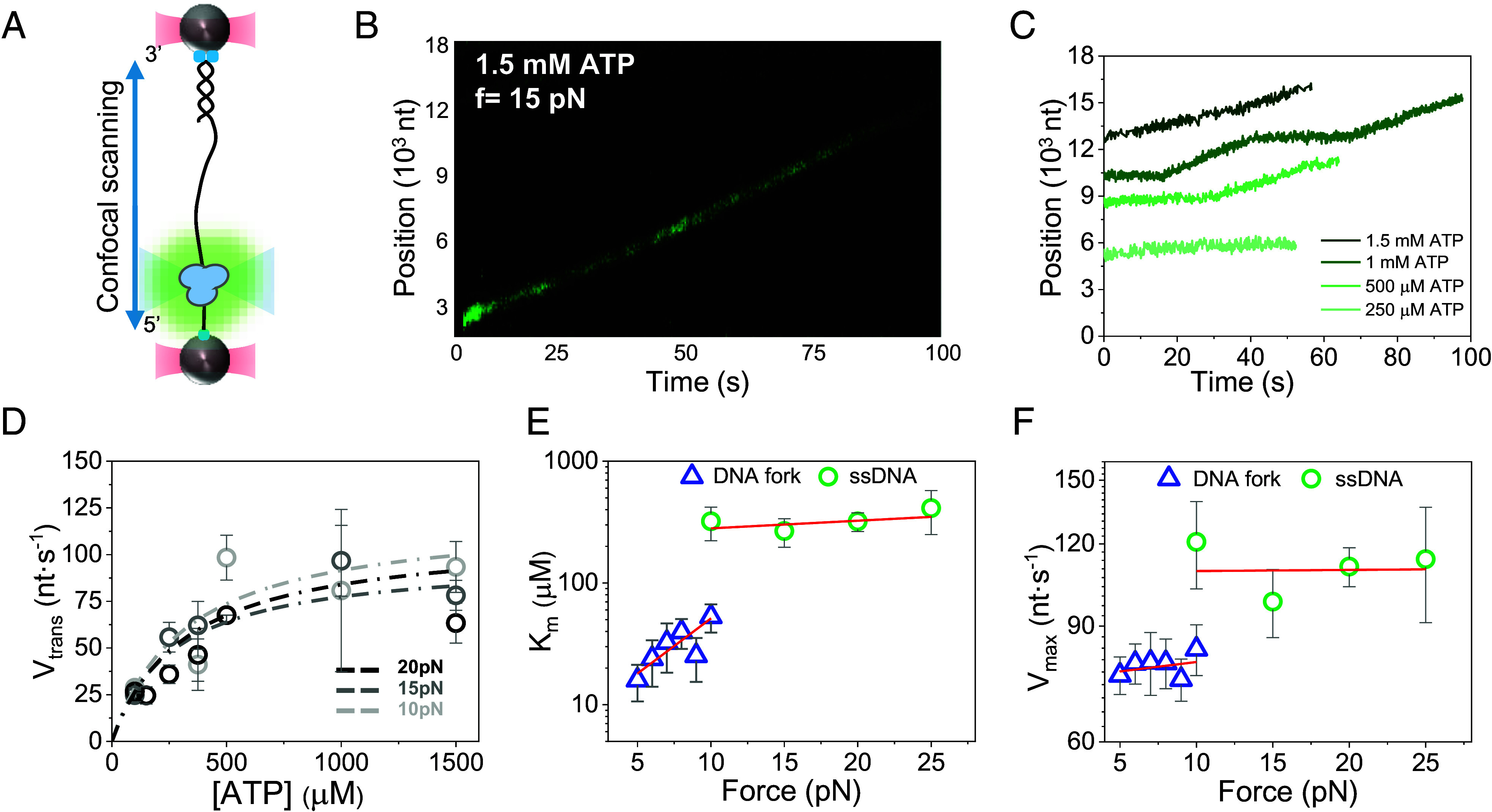
ATP concentration and force dependencies of Pfh1 translocation kinetics on ssDNA. (*A*) Diagram of the optical tweezers-confocal assay to image Alexa488(IgG)-Pfh1 (green) on individual ssDNA attached between two optically trapped beads (*Materials and Methods*). Pfh1 is expected to translocate with a 5′-3′ directionality. (*B*) Representative kymograph of a single Alexa488(IgG)-Pfh1 molecule (green) translocating directionally on ssDNA in the presence of ATP (*f* = 15 pN). (*C*) Representative position vs. time traces of Alexa488(IgG)-Pfh1 spots at increasing ATP concentrations (*f* = 15 pN). Traces were offset along the *Y*-axis for clarity of display. (*D*) ATP concentration dependencies of the pause-free ssDNA translocation velocity, *V_trans_*, under increasing force. Dash lines represent Michaelis–Menten fits to the data at different forces. (*E*) Force dependence of the apparent *K_m_* for ssDNA translocation (green symbols; linear fit, *m* = (6 ± 6) × 10^−3^) and DNA fork unwinding (blue symbols; fit using Eq. **1**, *Materials and Methods*). (*F*) Force dependence of *V_max_* for ssDNA translocation [green symbols; linear fit, m = (2 ± 2) × 10^−4^, *V_max_* = 109 ± 4 nt/s] compared with DNA fork unwinding (blue symbols; linear fit with slope m = 0.5 ± 0.7, *V_max_* = 75 ± 5 bp/s). For all data plots error bars show S.E.M.

DNA helicases unwind double-stranded DNA by coupling ATP hydrolysis to translocation along single-stranded DNA (ssDNA). To elucidate this coupling mechanism in Pfh1, we measured the force and ATP concentrations dependencies of the ssDNA translocation velocity of Pfh1. Using optical tweezers combined with confocal fluorescence microscopy (60), we tracked the position of Pfh1, labeled with anti-His tag Alexa488(IgG) (2 nM; *Materials and Methods*), as diffraction-limited spots on individual 18 kbp-long ssDNA molecules (Fig. 4*A* and *Materials and Methods*). Experiments were performed at forces greater than 10 pN to prevent ssDNA secondary structure formation, which could otherwise interfere with translocation.

Analysis of individual trajectories (*N* = 164, *SI Appendix*, Table S1) revealed that, in the presence of ATP, Pfh1 translocates unidirectionally along the ssDNA consistent with the 5′-to-3′ directionality of Pif1 helicases (61, 62). ssDNA translocation spanned thousands of nucleotides before the protein detached or photobleached (Fig. 4 *B* and *C*), which contrast with the limited (~20 bp) unwinding processivity, highlighting the impact of helicase–fork interactions on the helicase functionality.

The average and pause-free ssDNA translocation rates were force independent and increased hyperbolically with ATP concentration (Fig. 4*D* and *SI Appendix*, Fig. S8). Fitting the pause-free velocity (*V_trans_*) as a function of ATP concentration at various forces to the Michaelis–Menten equation (Fig. 4*D*) revealed a *K_m_* = 312 ± 19 μM and the *V_max_* = 109 ± 4 nt/s. Both values did not present significant force dependencies (Fig. 4 *E* and *F*) and were consistent with those obtained in previous single molecule and ensemble studies of Pif1 translocation on ssDNA (46, 49, 55, 61, 63, 64). While the *V_max_* for ssDNA translocation was ~1.3-times faster than for DNA unwinding, the apparent *K_m_* for ATP was ~six times higher on ssDNA than on the DNA fork at comparable forces (i.e., ~10 pN). The Michaelis constant (*K_m_*) increases with force during DNA unwinding but not during ssDNA translocation. Consequently, in the absence of force, the catalytic efficiency for ssDNA translocation (0.35 µM^−1^s^−1^) is predicted to be ~35-fold lower than that for DNA unwinding (12.5 µM^−1^s^−1^, *SI Appendix*, Fig. S5).

These results indicate that interaction with the fork increases the affinity of Pfh1 for ATP, favoring a more efficient coupling of chemical energy of ATP hydrolysis during unwinding than during translocation. This observation suggests that the helicase may adopt different conformations depending on the nucleic acid structure, as suggested previously for SF1 helicases (65–67).

### Rewinding Is Driven by ATP Hydrolysis.

**Fig. 5.**
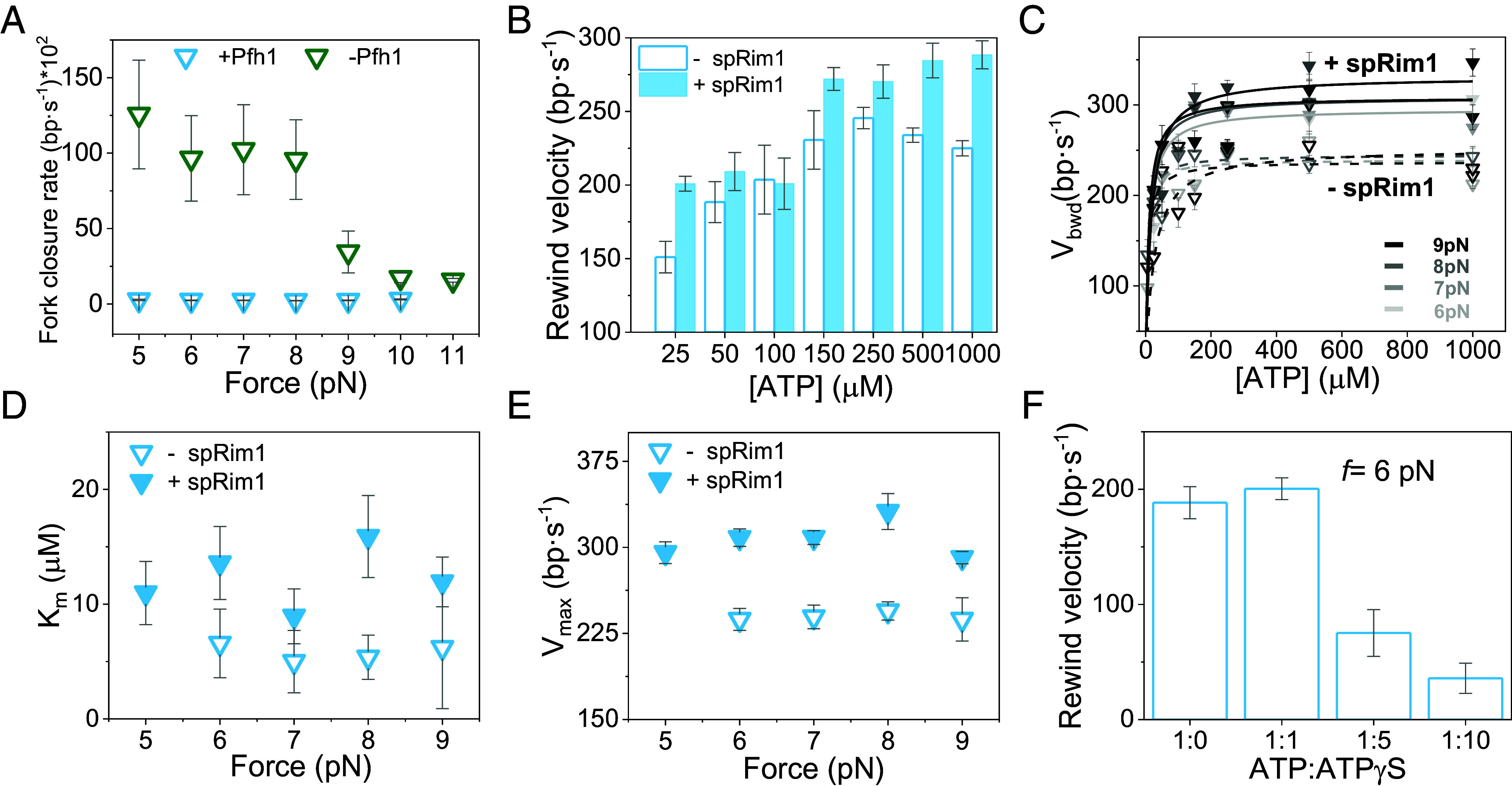
ATP and force dependencies of rewinding kinetics. (*A*) Spontaneous hairpin rezipping velocity in the absence of Pfh1 (green) and Pfh1-mediated rewinding velocity (blue) as a function of force. The hairpin rezipping velocity was measured in separate experiments by releasing the force after mechanical unzipping and observing the spontaneous rezipping in the absence of Pfh1 (*SI Appendix*, Fig. S9). (*B*) Average rewinding velocity as a function of ATP concentration in the absence (empty columns) and presence (filled columns) of spRim1 (2 nM). Average rewinding rates did not show significant force dependencies within the tested range (*SI* A*ppendix,* Fig. S10). Data were averaged and presented in (*B*) as the mean value at each ATP concentration for clarity of display (*N* = 5,190, *SI Appendix*, Table S1). (*C*) Backward rewinding velocity (*V_bwd_*) in the absence (open triangles) and presence (filled triangles) of spRim1 as a function of ATP concentration at different applied forces (gray scale). Data were fitted with the Michaelis–Menten equation (dash and solid lines). (*D* and *E*) Force dependencies of Michaelis–Menten parameters *K_m_* (*D*) and *V_max_* (*E*) for rewinding in the absence (open triangles) and presence of spRim1 (filled triangles). (*F*) Average rewinding rates as a function of the ATP:ATPγS ratio (*N* = 126). For all data plots error bars show the SEM.

The characteristic unwinding bursts of Pfh1 are followed by rewinding events that restore the hairpin to its original closed state (Fig. 1*C*). Pfh1-mediated rewinding velocities were force-independent, in sharp contrast to the strongly force-dependent spontaneous rezipping rate of a protein-free hairpin (Fig. 5*A* and *SI Appendix*, Fig. S9). Moreover, at the lowest forces, Pfh1-driven rewinding velocities were up to 50-fold slower than spontaneous hairpin rezipping (Fig. 5*A*). These results argue against helicase detachment upon unwinding and instead suggest that Pfh1 modulates rewinding velocity. We analyzed rewinding kinetics at forces below 10 pN, where the spontaneous rezipping and Pfh1-mediated rewinding rates were readily separable.

Interestingly, the average rewinding velocities (Fig. 5*B*), and the rewinding velocities after subtracting transient pauses and short forward events from each rewinding burst (backward velocity, *V_bwd_*, Fig. 5*B*) exhibited a hyperbolic increase with ATP concentrations across all forces (Fig. 5*C* and *SI Appendix*, Fig. S10), indicating that ATP binding and/or hydrolysis promotes rewinding. Remarkably, spRim1 (2 nM) stimulated the rewinding rates across all ATP concentrations and forces tested (Fig. 5 *B* and *C* and *SI Appendix*, Fig. S10). Fit of the backward rewinding velocities as a function of ATP concentration at varying forces with the Michaelis–Menten equation indicated that in the presence of spRim1 both the apparent *K_m_* for ATP and *V_max_* increased similarly by ~30 to 50% as compared to the absence of the SSB, Fig. 5 *C*–*E*. These results suggest that decreasing affinity for the nucleotide may result in a faster rewinding rate.

To determine the relationship between ATP binding/hydrolysis and rewinding activity, we measured the effect of the slowly hydrolyzable ATP analog ATPγS on rewinding kinetics. We performed these experiments at a fixed ATP concentration of 50 µM with increasing concentrations of ATPγS and at a constant force of 6 pN. The effects of the ATP analog on both unwinding (*SI Appendix*, Fig. S11) and rewinding (Fig. 5*F* and *SI Appendix*, Fig. S11) kinetics were only detectable at ATPγS:ATP ratios exceeding 1:1, suggesting its binding affinity is lower than that of ATP. At 5:1 and 10:1 ATPγS:ATP ratios, the rewinding velocity was reduced by up to ~25-fold compared to the velocity in the absence of the analog, Fig. 5*F*. This result indicates that ATP hydrolysis promotes while the NTP-bound state hinders rewinding velocity, probably by promoting more stable binding to DNA.

### Rewinding Activity Cannot be Explained by a Strand-Switching Mechanism.

**Fig. 6.**
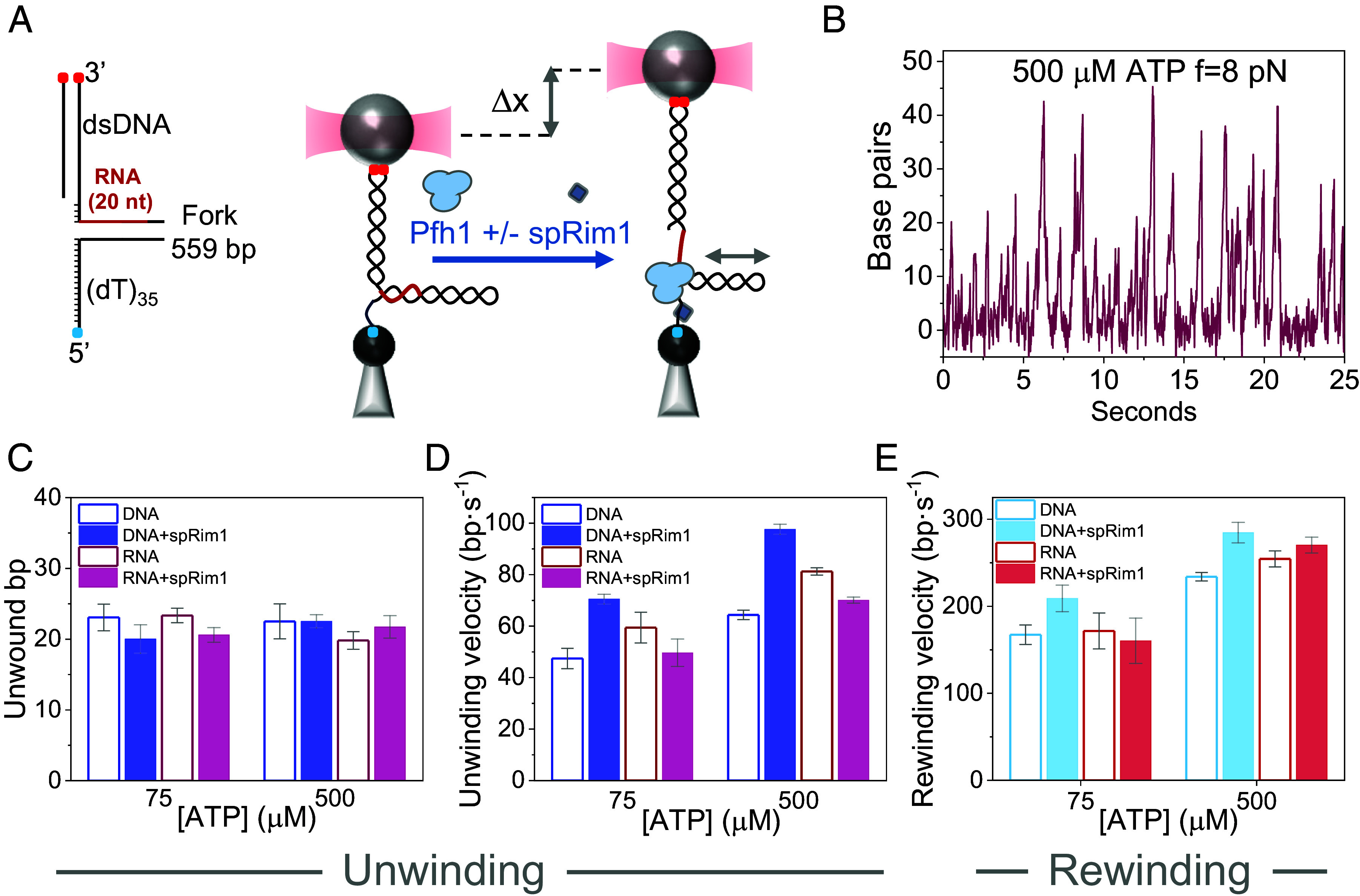
ATP and force dependencies of the unwinding/rewinding kinetics on a RNA–DNA fork. (*A*, *Left*) Schematics of the DNA/RNA hybrid fork. The RNA–DNA hairpin is identical to the DNA hairpin, except that the first 20 nt of the displaced strand (in red) were substituted with their RNA counterparts (*Materials and Methods* and *SI Appendix*, Table S2). (*Right*) Diagram of the optical tweezers assay to measure Pfh1 activities on the RNA–DNA fork in the absence or presence of spRim1. (*B*) Representative DNA unwinding/rewinding cycles of Pfh1 at the RNA–DNA hybri*d* fork *(f* = 8pN; 500 µM ATP). (*C* and *D*) ATP dependencies of the average number of unwound base pairs (*C*) and average unwinding velocity (*D*) on the RNA–DNA forks in the absence (empty magenta columns) and presence (filled magenta columns) of spRim1 (2 nM). For comparison, blue columns show the values measured in the DNA fork at equivalent ATP concentrations in the absence (empty columns) and presence (filled columns) of spRim1. (*E*) Average rewinding velocity of the RNA–DNA hybrid fork in the absence (empty red columns) and presence (full red columns) of spRim1. For comparison, blue columns show the values measured for the DNA fork at equivalent ATP concentrations. (*C*–*E*) Since kinetics did not show significant force dependencies within the tested range (*SI* A*ppendix,* Fig. S12), the data at different forces were averaged and presented as the mean value at each ATP concentration for clarity of display. For all data plots error bars show SEM.

The ATP dependent rewinding could be interpreted either as strand-switching, in which the helicase translocates on the displaced strand in the 5′-to-3′ direction away from the fork junction or, as a sliding back process in which ATP-dependent helicase–DNA interactions modulate the rewinding rate. To distinguish between these two possibilities, we measured the unwinding/rewinding kinetics of Pfh1 (2 nM) on a hybrid fork substrate in which the displaced strand consisted of RNA rather than DNA (*Materials and Methods* and Fig. 6*A*). Previous work showed that both Pif1 and Pfh1 efficiently unwind such RNA-containing hybrid substrates but do not translocate directionally on ssRNA (29, 31, 62, 68, 69). Therefore, we would expect differences between the rewinding kinetics on the DNA and RNA–DNA forks.

These experiments were performed at constant forces between 5 to 11 pN, ATP concentrations below (75 µM, *N* = 265, *SI Appendix*, Table S1) and above (500 µM, *N* = 627, *SI Appendix*, Table S1) saturation, and in the absence or presence of spRim1 (2 nM). Under these conditions, the unwinding/rewinding bursts for Pfh1 were hardly distinguishable between the RNA–DNA and the DNA forks at all forces (Fig. 6*B*). In the absence of spRim1, the average time between unwinding burst, unwinding processivity and velocity remained force independent for the RNA–DNA fork, as in the case of the DNA fork (*SI Appendix*, Fig. S12). Pfh1 displayed identical average unwinding processivities in both substrates (Fig. 6*C*), while the unwinding velocity on the RNA–DNA was ~20% faster than in the DNA fork (Fig. 6*D*). The latter observation agrees with the preference of Pif1 helicases for hybrid substrates (62, 68).

Remarkably, rewinding events were observed in both DNA and RNA–DNA forks with similar average rewinding velocities in both substrates across all forces and ATP concentrations (Fig. 6*E* and *SI Appendix*, Fig. S8). These results argue against strand switching, favoring the ATP-dependent sliding back hypothesis as a more plausible mechanism to explain the rewinding events.

Interestingly, in contrast with the results on the DNA fork, spRim1 did not stimulate the average unwinding and rewinding rates on the RNA–DNA fork at all forces (Fig. 6 *D* and *E* and *SI Appendix*, Fig. S12). Given that spRim1 is expected to bind more weakly to the displaced RNA strand (70–73), these results imply that spRim1 stimulates the unwinding and rewinding rates of Pfh1 on the DNA fork through an interaction with the displaced 3′ strand.

Finally, we note that in the presence of Pfh1, ~80% of RNA–DNA hairpins open at forces more than two standard deviations above the mean unzipping force measured in the absence of helicase (Fig. 2*G* and *SI Appendix*, Fig. S6). As described above, the same threshold was reached in ~31% of unzipping attempts in the DNA hairpin. The higher force requirement for the RNA–DNA hybrid suggests a more stable interaction with RNA than with DNA in the displaced strand. Interestingly, spRim1 restored the unzipping force close to the value in the absence of the helicase in the DNA hairpin but not in the RNA–DNA hairpin, where it remained unaltered (Fig. 2*G*). These observations imply that spRim1 destabilizes the interaction of Pfh1 with the displaced strand in the DNA hairpin.

## Discussion

Using optical tweezers and single-molecule imaging, we show that a single Pfh1 helicase unwinds DNA through successive cycles of unwinding and rewinding bursts, whose kinetics are tightly regulated by helicase–DNA interactions at the replication fork. Here, we identify key determinants of strand separation, define the intrinsic limits of unwinding processivity, and reveal how ATP-dependent rewinding and partner proteins modulate Pfh1 activity at the fork.

Our results show that the average unwinding processivity of Pfh1 (~20 bp) did not correlate with unwinding velocity (i.e., faster rates did not produce longer unwound tracts) and was independent of force, helicase, and ATP concentrations, RNA content in the displaced strand, and spRim1 binding. These observations indicate that processivity is an intrinsic property of the helicase at the fork rather than a stochastic time-dependent process. What, then, constrains the intrinsic processivity? The increased force required to mechanically unzip DNA and RNA–DNA hairpins in the presence of Pfh1 suggests that the helicase forms stable contacts with both strands at the fork, thereby protecting it from mechanical unfolding. In support of this idea, secondary DNA-binding interactions have been proposed for Pfh1 and other helicases (30, 52, 74–76). We therefore speculate that during unwinding, persistent engagement of Pfh1 with both fork strands may alter the local fork geometry and thereby influence helicase–DNA interactions that ultimately limit forward translocation and define the characteristic unwinding processivity of the helicase.

Furthermore, our results indicate that the catalytic efficiency during unwinding was ~35-fold higher than during ssDNA translocation, whereas processivity was at least two orders of magnitude shorter. These observations reinforce the idea that engagement with both DNA strands is essential for productive unwinding but simultaneously limits overall processivity. Therefore, the displaced strand would play the role of a functional interaction partner imposing geometric constraints and/or additional binding free energy that modulate helicase progression. Depending on their configuration, these contacts may either stabilize forward unwinding or act as a “mechanical brake” that stalls the helicase. This model of regulation would be relevant for the essential and nonredundant role of the multifunctional Pfh1. Consistent with this view, interactions with the displaced strand have been shown to regulate the activity of several other helicases (52, 76).

The enhanced coupling between ATP hydrolysis and unwinding further suggests that Pfh1 adopts distinct “functional conformations” depending on the nucleic acid substrate (65–67) implying that direct comparisons between unwinding and translocation velocities may not provide a reliable measure of helicase “activeness” (77) within this family.

The average unwinding velocity of Pfh1 depended on ATP concentration, as expected for a molecular motor. Michaelis–Menten analysis revealed that applied force increases the apparent ATP affinity constant *K_m_* without affecting *V_max_*. As a consequence, force-dependent effects on velocity were observed only under subsaturating ATP conditions. These results indicate that ATP binding, and its associated conformational changes, are not rate limiting at saturating ATP concentrations.

Arrhenius analysis of *K_m_*(*f*) indicates that the force-dependent decrease in ATP affinity corresponds to a conformational change of ~0.8 nm, consistent with release of at least two nucleotides (∼0.34 nm/nt) from the translocating strand along the pulling coordinate. Because in related Pif1 helicases ATP binding modulates the affinity of the enzyme for the translocation strand (69), while binding to the translocation strand in turn regulates ATP turnover (78) perturbation of these contacts by external force provides a direct explanation for the observed increase in *K_m_*(*f*) and reduction in catalytic efficiency under load.

We note that, in stark contrast, force has been shown to positively affect both processivity and unwinding velocity of the *S. cerevisiae* homolog Pif1 (37), suggesting that even within the same helicase class, there are different modalities of activity and potential regulatory mechanisms.

Our results indicate that spRim1 binding to the displaced DNA strand facilitates mechanical unzipping by the applied force and enhances unwinding velocity. We propose that this effect arises from competition between spRim1 and a secondary nucleic-acid–binding site of Pfh1 for the nontranslocating strand exposed during unwinding, which otherwise slows helicase progression. In addition, spRim1 reduced the time interval between unwinding bursts at all ATP concentrations. Because the short 4-nt 3′ tail is unlikely to support stable spRim1 binding during initiation, these results suggest that interaction of spRim1 with the 5′ strand (30-nt) stabilizes Pfh1 in a productive unwinding conformation at the fork.

In Pif1 and related helicases, ATP-dependent rewinding has been interpreted as evidence for strand-switching (37, 41, 42). Here, we show that rewinding velocity is independent of force, substantially slower than spontaneous hairpin rezipping, faster than ssDNA translocation, and dependent on ATP concentration and hydrolysis. These observations support an active regulation of rewinding by Pfh1. However, because Pfh1 displays similar rewinding kinetics on DNA and RNA–DNA forks, despite the inability of Pif1-family helicases to translocate on ssRNA (62, 69), our results argue against strand switching as a general mechanism for rewinding.

Instead, we propose that upon stalling, Pfh1 transiently loosens its grip on the translocating strand, allowing the free-energy gain associated with DNA reannealing at the fork to drive backward motion and initiate rewinding. Importantly, ATP binding and hydrolysis modulate this process by regulating Pfh1–DNA contacts, as supported by the pronounced slowing of rewinding in the presence of ATPγS. Within this framework, the counterintuitive ability of spRim1 to accelerate rewinding on DNA but not RNA–DNA forks, is consistent with spRim1 interacting with the displaced DNA strand and competing with helicase–DNA interactions. Such a sliding-back rewinding mechanism is likely relevant to the role of Pfh1 in promoting replisome progression (17, 26) by preventing head-on collisions with the leading-strand polymerase, thereby preserving the structural integrity of the replisome. We note that while our results do not support strand switching as a general mechanism for fork rewinding, we cannot exclude its occurrence on different substrates, such as G-quadraplexes-containing constructs (42).

**Fig. 7.**
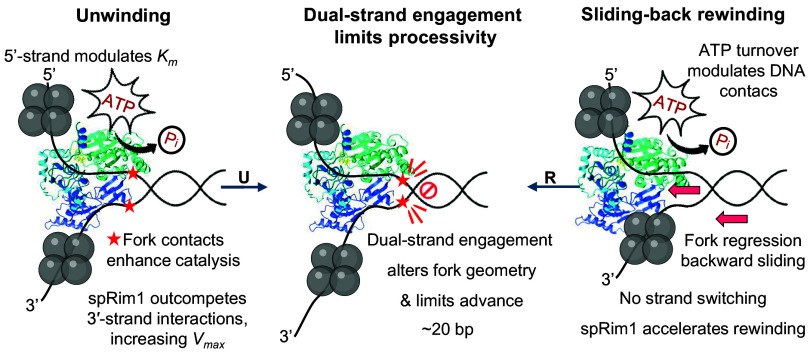
Proposed model for the coordinated unwinding and rewinding activities of Pfh1 and their regulation by fork interactions and spRim1.

In conclusion, our results support a model in which helicase-mediated DNA unwinding is regulated by a sliding-back rewinding mechanism, Fig. 7. During unwinding, the catalytic efficiency of Pfh1 is enhanced through coordinated interactions with both DNA strands at the fork: Contacts with the translocating strand modulate ATP affinity, whereas interactions with the displaced strand limit *V_max_*. Binding of spRim1 to the displaced strand disrupts the later interactions and increases unwinding velocity. We propose that stable engagement of Pfh1 with both strands at the fork alters the local fork geometry, thereby modifying helicase–DNA interactions in a way that limits forward translocation and defines the intrinsically short unwinding processivity. Once this limit is reached, transient loosening of helicase contacts with the translocating strand in an ATP-dependent state allows fork regression to drive backward motion and initiate rewinding without strand switching or dissociation. Continued ATP hydrolysis modulates helicase–DNA contacts during this sliding-back phase, while spRim1 further accelerates rewinding by competing for displaced-strand interactions. Together, these results support a mechanochemical model in which Pfh1 activity is dynamically balanced between unwinding and rewinding through coordinated strand-specific interactions and regulation by partner proteins.

## Materials and Methods

### DNA Constructs for Single Molecule Experiments.

The DNA hairpin construct consists of a 2,686 base pairs (bp) DNA “handle” (pUC19 vector, Novagen) labeled with digoxigenin at one end, a 5’ (dT)_35_ end functionalized with biotin, and a 559 bp stem capped by a (dT)_4_ loop (79). The hairpin stem sequence is shown in *SI Appendix*, Table S1. The RNA–DNA hairpin is identical to the DNA hairpin, except that the first 20 nt of the displaced strand (in red) were substituted with their RNA counterparts.

The ssDNA tether was formed by tethering between two streptavidin-coated polystyrene beads the biotinylated ends of a 17,303 bp dsDNA that was nicked at two positions separated by 14,987 nt within the same strand (80). Application of force higher than 80 pN to tether promoted dissociation of the nicked strand and yielded a hybrid DNA tether containing 14,987 nt of ssDNA and 2,136 bp dsDNA.

### Buffers and Proteins.

Single molecule studies were carried out in the reaction buffer containing 20 mM Tris pH 7.5, 30 mM KCL, 1 mM DTT, 4 mM MgCl_2_, 0.2 mg/mL BSA, and ATP concentrations ranging from 5 μM to 1.5 mM. *S. pombe* Pfh1 and SpRim1 were overexpressed and purified as previously described (31, 81).

### Single-Molecule Force Spectroscopy Experiments and Data Analysis.

We manipulated individual DNA hairpins using a miniaturized dual-beam optical tweezers instrument (57). Each hairpin was tethered between a streptavidin-coated bead (2.1 μm, Kisker Biotech) immobilized on a micropipette and an anti-digoxigenin-coated bead (3.0 μm, Kisker Biotech) held in the optical trap. Proteins were introduced into the flow cell after dilution in the reaction buffer. Unless otherwise indicated, assays contained 2 nM Pfh1 and 2 nM spRim1. Consistent unwinding activity required ATP; concentrations of ATPγS exceeding ten times the ATP concentration failed to support unwinding.

In all cases, data were collected at 500 Hz at 22 ± 1 °C using a feedback loop to maintain a constant force on the DNA. The range of applied forces was from 5 to 11 pN, a regime at which the dsDNA hairpin remains fully paired. The trap stiffness calibrated for 3.0 μm beads was *k* = 0.135 ± 0.0043 pN nm^−1^.

The number of unwound base pairs was determined by dividing the total change in extension in each unwinding burst (i.e., from the beginning of the activity to the maximum extension unwound in each activity burst) by the force-dependent extension increase associated with each catalytic step, which generates two new ssDNA nucleotides (52). We used the average extension per nucleotide as a function of force reported previously for ssDNA measured under similar experimental conditions (82).

The mean unwinding and rewinding rates were calculated by dividing the total number of unwound/rewound base pairs by the time to accomplish each unwinding or rewinding event.

Forward unwinding (*V_fwd_*) and backward rewinding (*V_bwd_*) velocities were determined using the PLANT (Piecewise Linear Approximation of Noisy Trajectories) algorithm (83), which approximates single-molecule trajectories as a series of linear segments identified by changes in slope (*SI Appendix*, Fig. S4). Segments corresponding to directed motion were selected based on the sign and magnitude of their slopes. For unwinding, segments with positive slopes larger than the mean unwinding velocity were retained, whereas for rewinding, segments with negative slopes larger in magnitude than the mean rewinding velocity were selected. Consecutive qualifying segments were merged, and velocities were computed from the total displacement and elapsed time of the merged intervals.

The average time between consecutive unwinding events was calculated as the difference between the start time of the unwinding activity of a given burst and the end time of the rewinding activity of the preceding burst.

We considered that the apparent affinity for ATP, *K_m_*, follows an Arrhenius force dependency (59),

[1] Kmf=Km0efdkBT,

where *K_m_*(0) corresponds to the value of apparent affinity for ATP in the absence of force (*f*) and *d is* the effective distance of the conformational change associated with the process.

For a helicase that exhibits a kinetic step size of 1 bp, *V_max_* can be approximated to catalytic rate, *k_cat_*, at the single molecule level. Therefore, the catalytic efficiency, *V*_max_*/K*_m_(*f*) adopts the following expression

[2] $$\frac{V_{\max}}{K_{m}\left(f\right)}=\frac{V_{\max}}{K_{m}\left(0\right)e^{fd/(k_{B}T)}}.$$

### Single-Molecule Fluorescence Imaging and Data analysis.

Pfh1 with a N-terminal 6×-His tag was labeled by binding it to an anti-6×-His Tag monoclonal antibody, conjugated to an Alexa 488 Fluorophore [Alexa488(IgG)-Pfh1]. The movement of Alexa488(IgG)-Pfh1 along the ssDNA was monitored by holding the ssDNA tether under force.

Single-molecule imaging experiments of Alexa488(IgG)-Pfh1 were performed on an instrument that combines three color confocal fluorescence microscopy (488, 532, and 635 nm), with optical tweezers and microfluidics (C-trap® LUMICKS). Trap stiffness was adjusted to 0.32 pN/nm in both traps. DNA-molecules were trapped between two streptavidin-coated polystyrene beads (4.38 μm diameter). The tethering of single DNA molecules was confirmed by analyzing the force extension curve. The DNA was then transferred to another channel (channel 4), previously passivated with BSA (2 mg/mL), and the distance between both beads was fixed to achieve a force of 10, 15, and 20 pN, and then Alexa488(IgG)-Pfh1 (2 nM) was flowed into the channel. We used the 488 nm excitation laser for visualization of Alexa488(IgG)-Pfh1 (emission filter of 500 to 525 nm). Kymographs were generated by single-line scans covering the entire DNA stretched between the two beads and the edge of both beads. Pixel size was set to 100 nm and the illumination time per pixel was 0.1 ms, resulting in a typical time per line of 28.1 ms.

We used the greedy algorithm (84–86) implemented in Pylake Python library (https://github.com/lumicks/pylake; DOI: 10.5281/zenodo.4280788) to track the position (µm) of individual fluorescent spots with time (s). Kymograms were aligned using the position of the beads as fiducial points. The distance in µm was converted to single-stranded nucleotides (nt) using the theoretical Freely Jointed Chain model of polymer elasticity. We used the interp1 MATLAB function to interpolate data in time and position.

The average translocation velocity was determined by dividing the total translocation length of the trace by the time that the helicase spends moving. The pause-free translocation velocity was determined as previously described (43, 87). Briefly, individual trajectories were analyzed with an algorithm that computes the instantaneous velocities, averaging the position over sliding time windows.

## Supplementary Material

Appendix 01 (PDF)

## Data Availability

Optical Tweezers data; values of changes in distance vs time; data have been deposited in (88).
