# Supplementary material for: Regulation of Pfh1 helicase activity by nucleic acid interactions and mitochondrial SSB
Source: Proc Natl Acad Sci U S A. 2026 May 18;123(21):e2602528123. doi: 10.1073/pnas.2602528123 (PMC13213944; doi:10.1073/pnas.2602528123)
Supplement: Supplementary file 1 — Appendix 01 (PDF) [file pnas.2602528123.sapp.pdf]

PNAS

Supporting Information for

Regulation of Pfh1 helicase activity by nucleic acid interactions and mitochondrial SSB.

María Ortiz-Rodríguez[a], Saurabh P. Singh[b], Francisco J. Cao-García[a,c], Roberto Galletto[b,1], Borja Ibarra[a,d,1]

[a] Instituto Madrileño de Estudios Avanzados en Nanociencia, IMDEA Nanociencia, Madrid, Spain.
[b] Department of Biochemistry and Molecular Biophysics, Washington University School of Medicine, St. Louis, MO 63110, USA.
[c] Departamento de Estructura de la Materia, Física Térmica y Electrónica, Universidad Complutense de Madrid, Pza. de Ciencias, 1, 28040 Madrid, Spain.
[d] Nanobiotecnología (IMDEA-Nanociencia), Unidad Asociada al Centro Nacional de Biotecnología (CSIC), Madrid, Spain.

[1]corresponding authors: Roberto Galleto, Borja Ibarra.

galletto@wustl.edu; borja.ibarra@imdea.org

This PDF file includes:

Figures S1 to S12

Tables S1 to S2

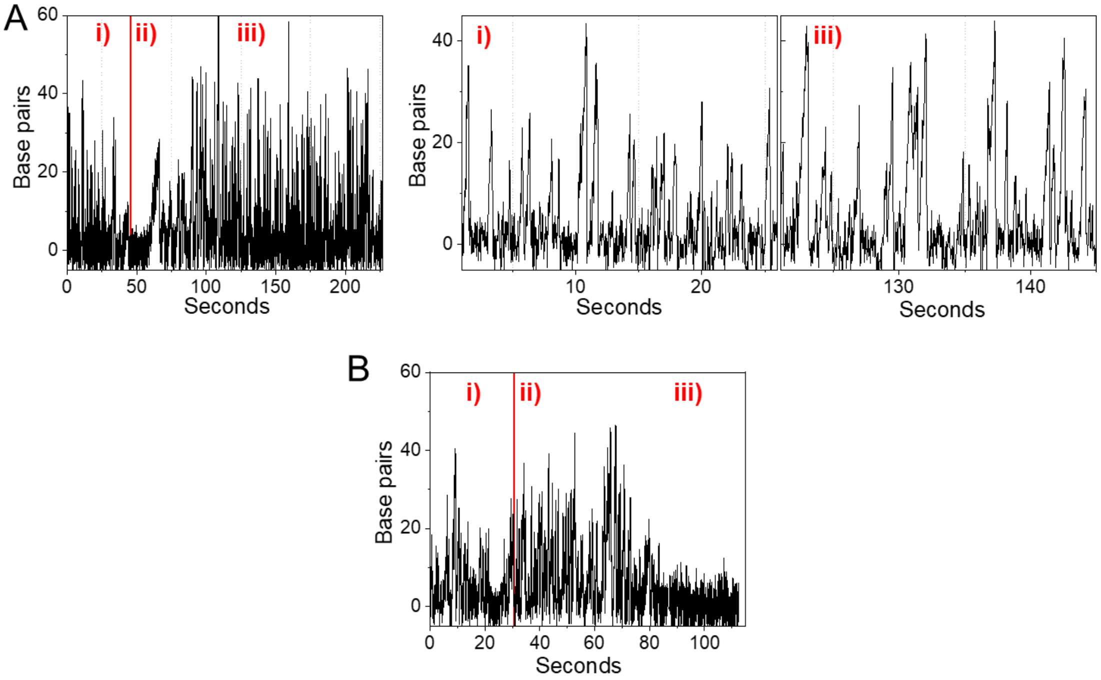

Figure S1. Persistence of Pfh1 unwinding–rewinding bursts after removal of free helicase from solution. A) A single DNA hairpin tethered between two trapped beads was held at constant tension while Pfh1 activity was recorded. (Left) Upon detection of unwinding-rewinding bursts (i), the chamber was gently flushed with a helicase-free reaction buffer containing ATP and $MgCl_2$ (ii, red line), thereby maintaining catalytic conditions while removing free Pfh1 from solution. Under these conditions, unwinding–rewinding bursts persisted for several minutes after washing the chamber (iii). (Right) Zoom in of unwinding bursts before (i) and after (iii) washing helicase from solution. **B)** Control experiment used to determine the time required to replace the buffer surrounding the optical trapping region. Upon detection of unwinding-rewinding activity (i), the chamber was flushed with a helicase-free reaction buffer lacking ATP and $MgCl_2$ (ii, red line). In this case, helicase activity ceased after 45±5 s (N=5), indicating the time required for the new buffer to reach the trapping region (iii). A) and B) Both experiments were performed under identical force (7 pN) and flow conditions. These experiments demonstrate that unwinding–rewinding bursts persist long after free helicase has been removed from solution, indicating that the repetitive cycles arise from the activity of a single Pfh1 molecule that remains bound to the DNA rather than to repeated binding events from solution.

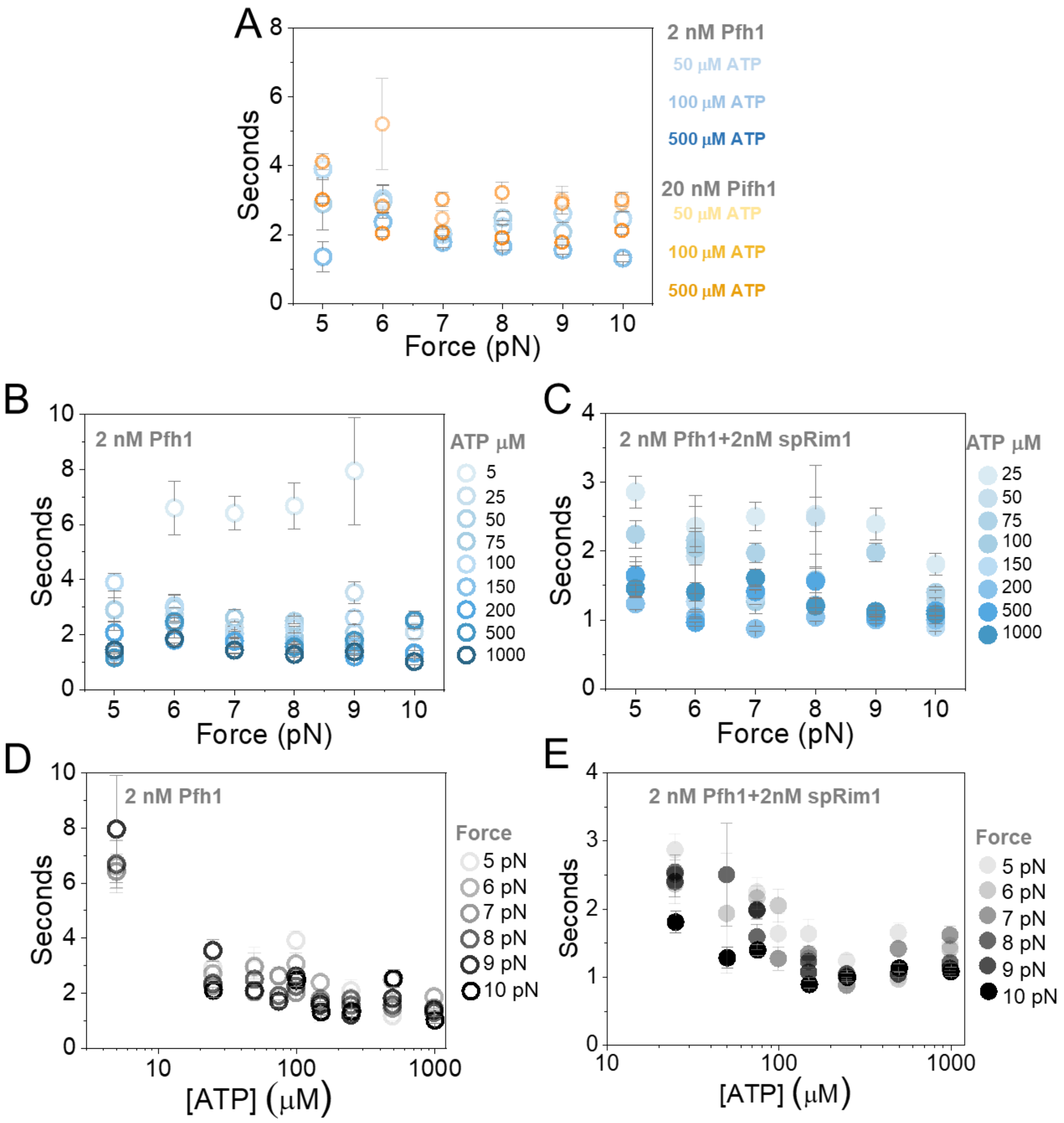

Figure S2: Average time between consecutive unwinding events. A) Average time between unwinding burst as a function of applied force, measured at 2nM (blue) or 20nM Pfh1 (orange) and different ATP concentrations. **B)** and **C)** Average time between unwinding burst as a function of applied force, measured at different ATP concentration in the absence (B) and presence (C) of 2nM spRim1. **D)** and **E)** Average time between unwinding burst as a function of ATP concentration, measured at different constant forces in the absence (D) and presence (E) of 2nM spRim1. In all panels, data points represent the mean, and error bars indicate the standard error of the mean (s.e.m.).

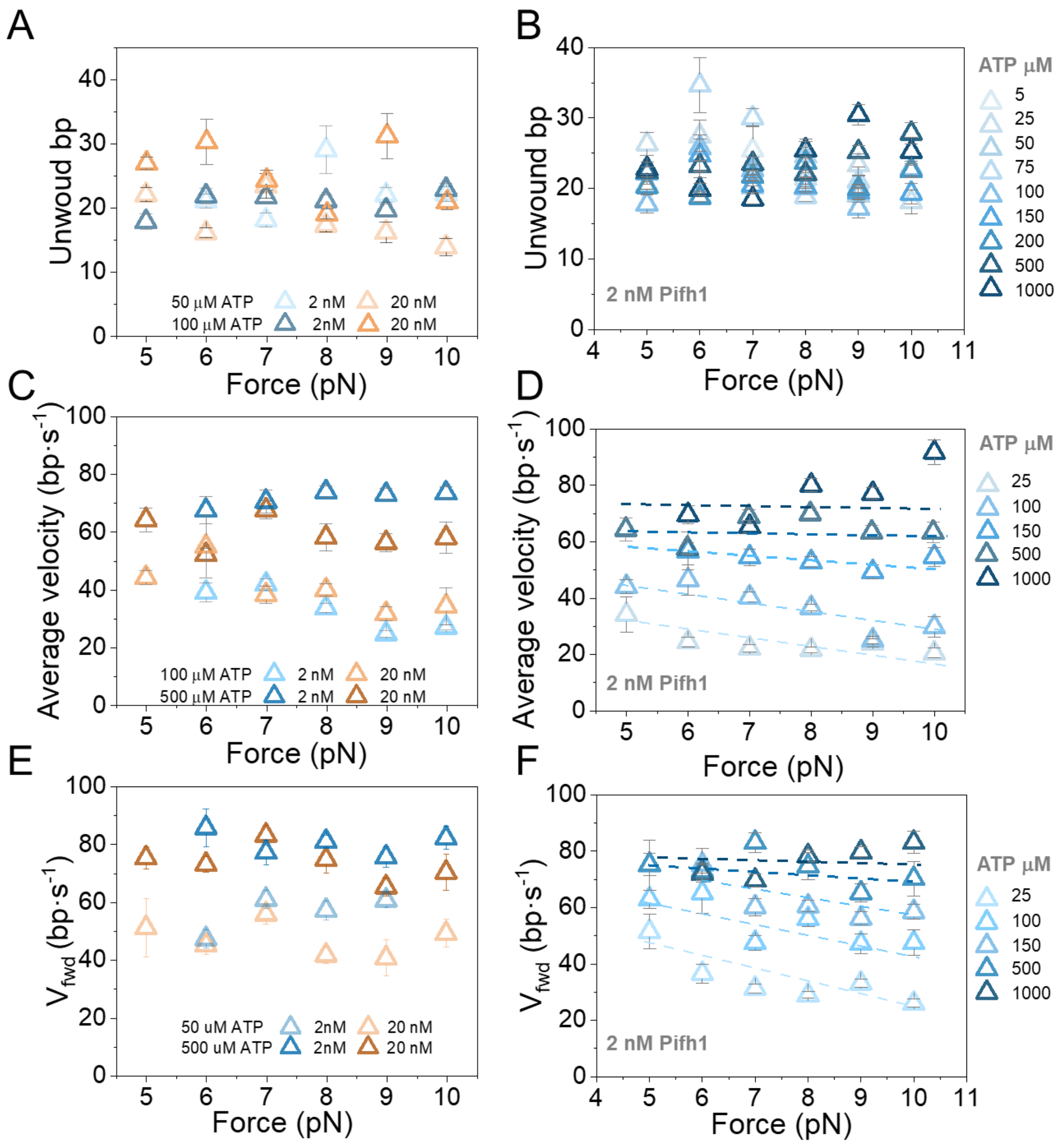

Figure S3: Effects of force and ATP concentration on the unwinding kinetics. A) and **B)** average number of unwound base pairs as a function of the applied force, measured at varying helicase (A) and ATP (B) concentrations. **C)** and **D)** Average unwinding velocity as a function of the applied force, measured at varying helicase (C) and ATP (D) concentrations. **E)** and **F)** Pause-free unwinding velocity as a function of applied force, measured at varying helicase (E) and ATP (F) concentrations. Dashed lines are shown as a guide to the eye and do not represent fits. Error bars represent the s.e.m.

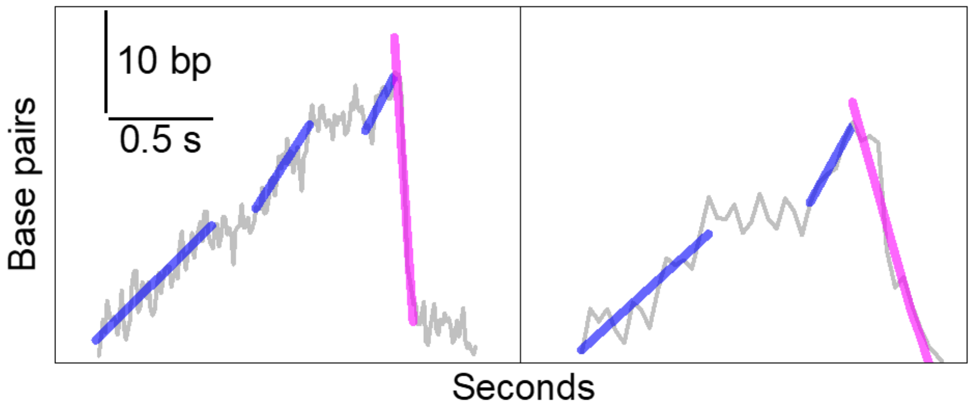

Figure S4: Calculation of unwinding burst velocities using the PLANT algorithm. This algorithm approximates a single-molecule trajectory (shown in grey) as a series of linear segments, identified by changes in slope (Left panel, 500 μM ATP. Right panel, 50 μM ATP. *f*= 6 pN). Segments representing directed motion were selected based on the sign and magnitude of their slopes: positive slopes greater than the mean unwinding velocity defined the unwinding burst velocity (blue), while negative slopes whose magnitude exceeded the mean rewinding velocity defined rewinding bursts (magenta). Consecutive qualifying segments were merged, and velocities were calculated as the total displacement divided by the elapsed time for each merged interval.

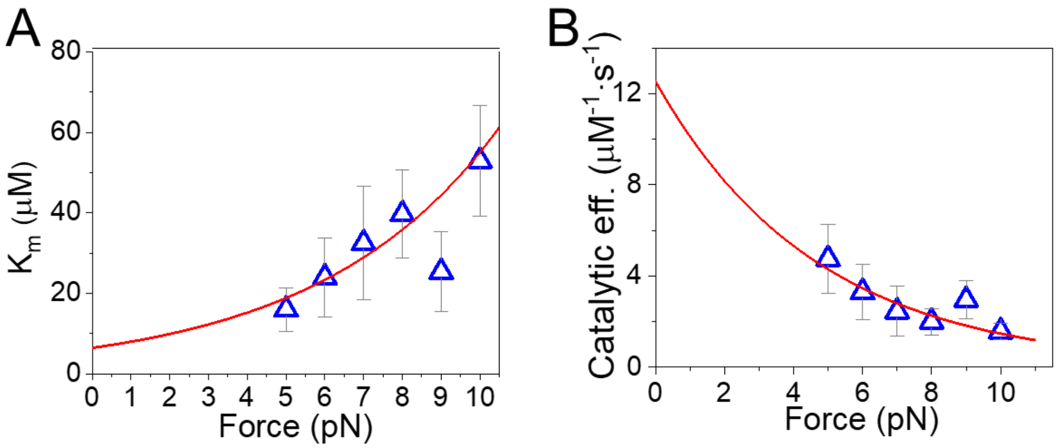

Figure S5: Force dependence of Pfh1's apparent ATP affinity and catalytic efficiency on a DNA fork substrate: A) Apparent Michaelis constant (K_m) for ATP as a function of applied force. The red line represents a fit to eq. 1 (Methods). **B)** Catalytic efficiency as a function of applied force. The red line represents a fit to eq. 2 (Methods). In both panels, error bars indicate the s.e.m.

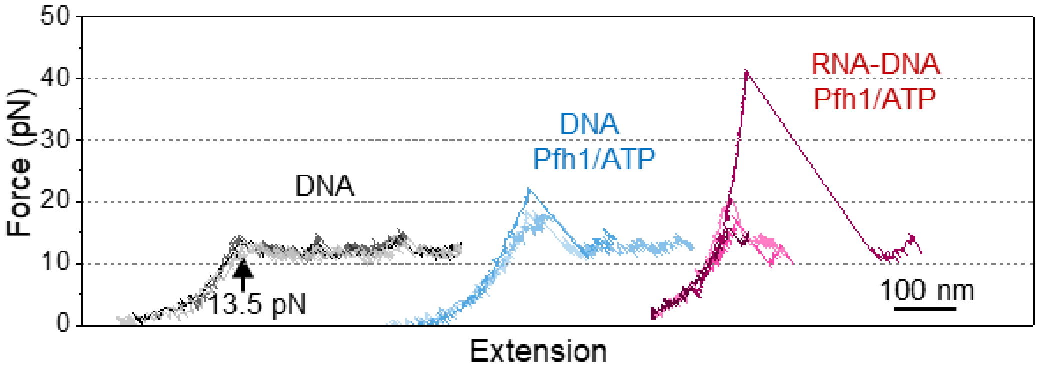

Figure S6: Unzipping force measurements. Initial sections of representative force-extension curves for the DNA hairpin (black); the DNA hairpin in the presence of 2 nM Pfh1 and 500 µM ATP (blue); and the RNA hairpin in the presence of 2 nM Pfh1 and 500 µM ATP (magenta). Three curves are shown per experimental condition. Arrow indicates the mean initial unzipping force without helicase, *f*~ 13.5 pN (*N*= 22).

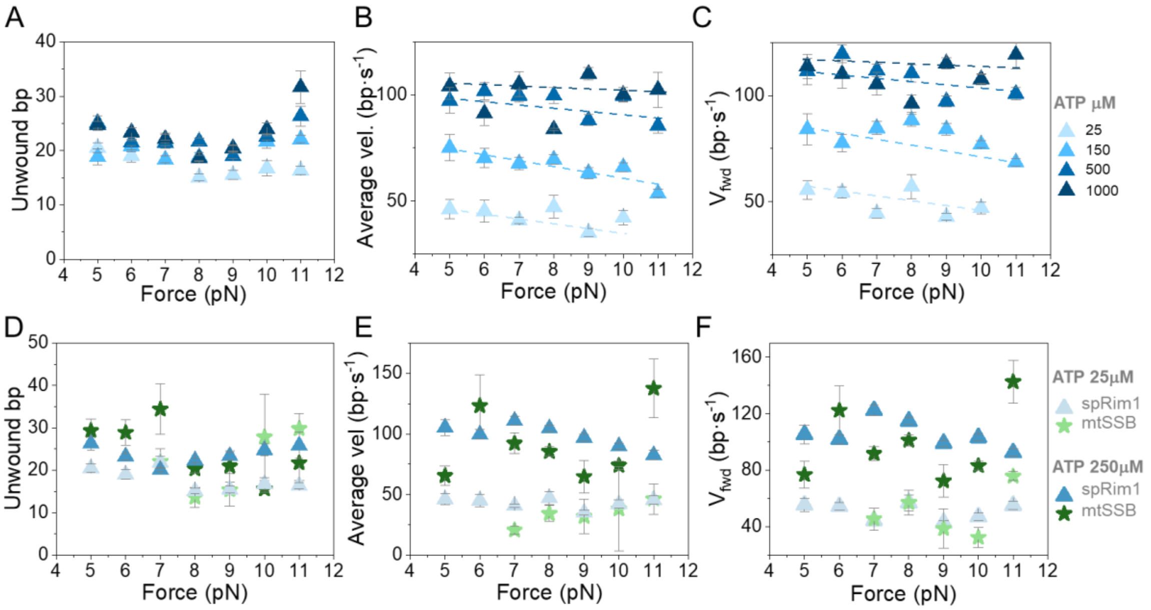

Figure S7: Effects of force and ATP concentration on the unwinding kinetics in the presence of spRim1 or mtSSB. A), B) and C) Average number of unwound base pairs (A), unwinding velocity (B) and pause-free velocity (C) of Pfh1 (2nM) as a function of the applied force and in the presence of spRim1 (2 nM), measured at varying ATP concentrations. Dashed lines are shown as a guide to the eye and do not represent fits. **D), E) and F)** Average number of unwound base pairs (D), unwinding velocity (E) and pause-free velocity (F) of Pfh1 (2nM) as a function of the applied force and in the presence of spRim1 (2 nM), or mtSSB (2 nM) proteins, measured at 25 and 250 □M ATP. For all plots, error bars are the s.e.m.

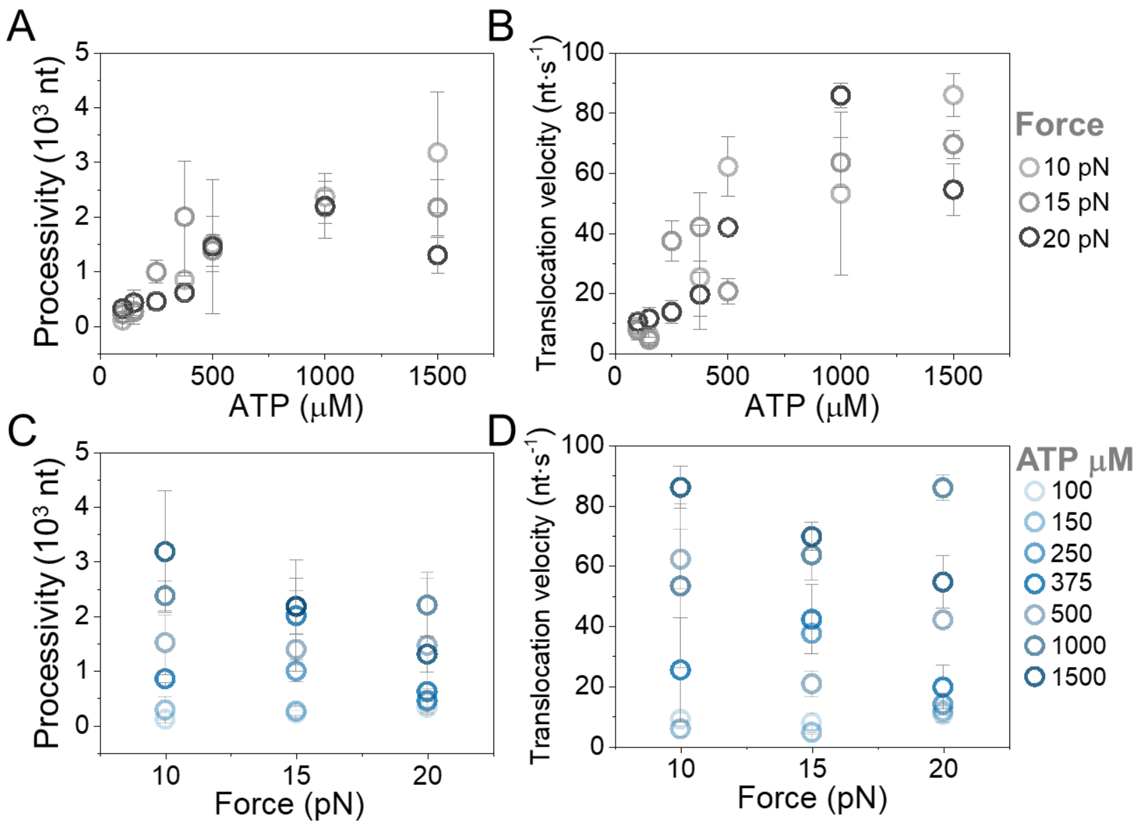

Figure S8: ATP concentration and force dependencies of Pfh1 ssDNA translocation kinetics. A) and B) Average processivity (A) and ssDNA translocation velocity (B) as function of ATP concentration, measured for different constant forces. **C) and D)** Average processivity (C) and ssDNA translocation velocity (D) as function of applied force, measured for varying ATP concentrations. For all plots, error bars show the s.e.m.

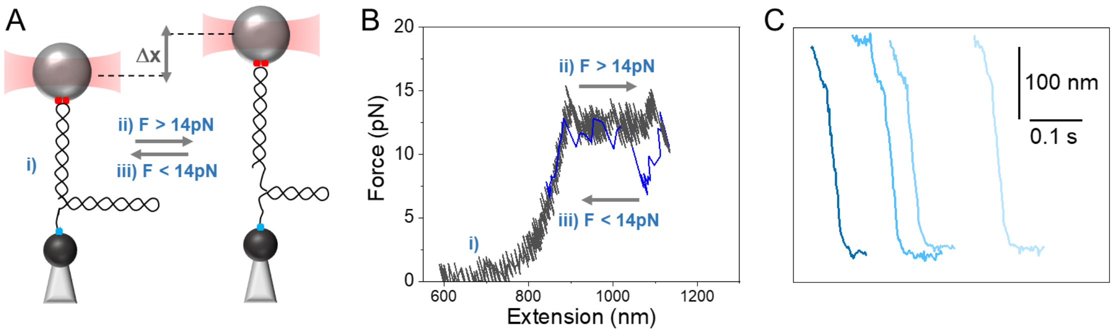

Figure S9: Determination of the spontaneous hairpin rezipping velocity in the absence of Pfh1. (A) Experimental design. (i) A single DNA hairpin construct was tethered between two functionalized microspheres, as described in the main text. (ii) Mechanical force above 14 pN was applied to the complementary strands to partially unzip the hairpin region of the DNA construct. (iii) The applied force was then reduced and kept constant below 14 pN. Under these conditions, the DNA hairpin spontaneously rezips. **B)** Representative force–extension curve showing (i) the initial force-extension curve, (ii) partial mechanical unzipping of the DNA hairpin above 14 pN and (iii) rapid drop in force to 7 pN accompanied by the subsequent spontaneous rezipping of the DNA (blue). **C)** Representative rezipping traces (f=7 pN), from which the spontaneous rezipping velocity was determined (traces were shifted along the time axis for clarity of display).

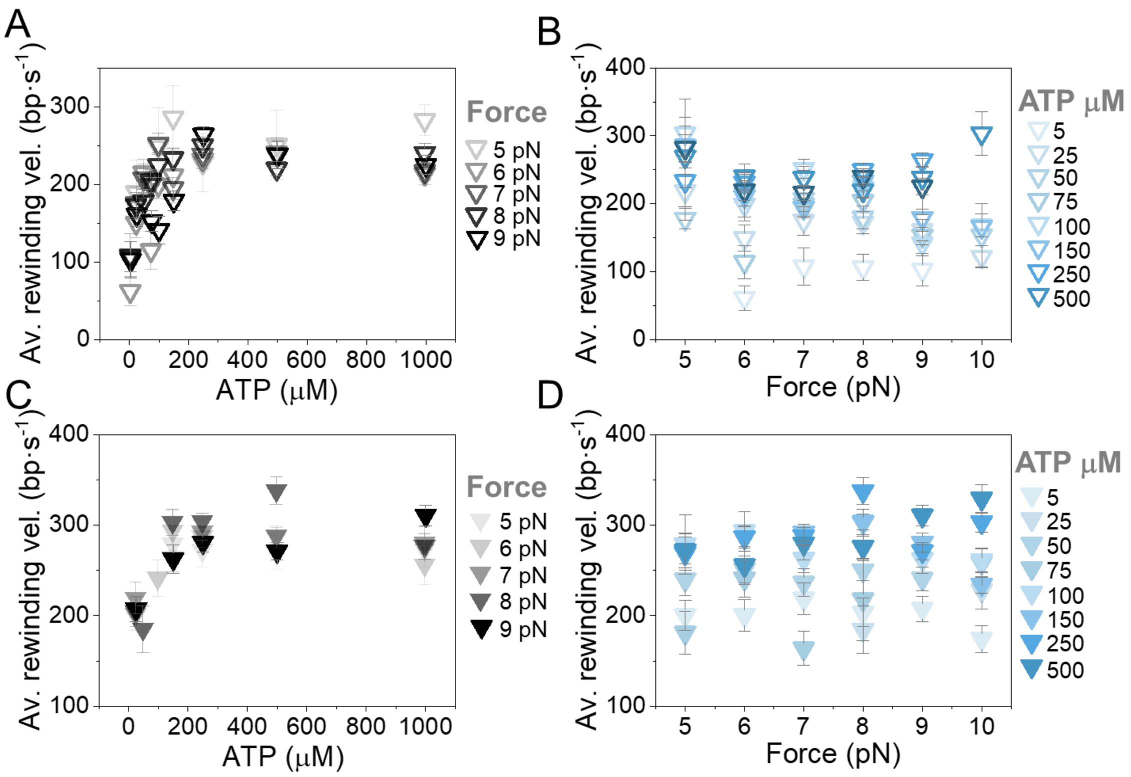

Figure S10: ATP concentration and force dependencies of Pfh1 rewinding velocity in the absence and presence of spRim1. A) Average rewinding velocity as a function of ATP concentration, measured for varying constant forces in the absence of spRim1. **B)** Average rewinding velocity as a function of applied constant force, measured for different ATP concentrations in the absence of spRim1. **C)** Average rewinding velocity as a function of ATP concentration, measured for varying forces in the presence of spRim1 (2nM). **B)** Average rewinding velocity as a function of applied force, measured for different ATP concentrations in the presence of spRim1. For all plots, error bars show the s.e.m**.**

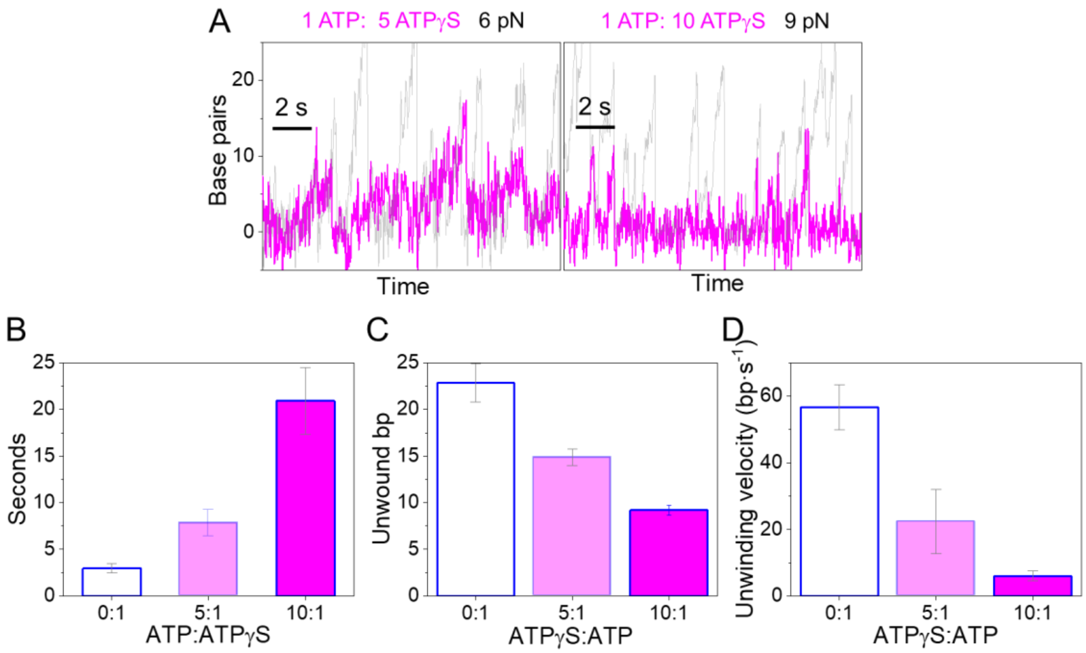

Figure S11: Effect of ATPγS on the unwinding kinetics: A) Representative DNA unwinding/rewinding cycles (in magenta) measured at 1:5 (left, f= 6 pN) and 1:10 (right, F= 9 pN) ATP: ATPγS ratios (50 µM ATP). For comparison, representative traces at equivalent forces and ATP concentrations in the absence of ATPγS are shown in light grey. **B)** to **D)** Effect of varying ATP:ATPγS ratios on the average time between DNA unwinding bursts (B), unwinding processivity (C) and unwinding velocity (D). For all plots, error bars show the s.e.m.

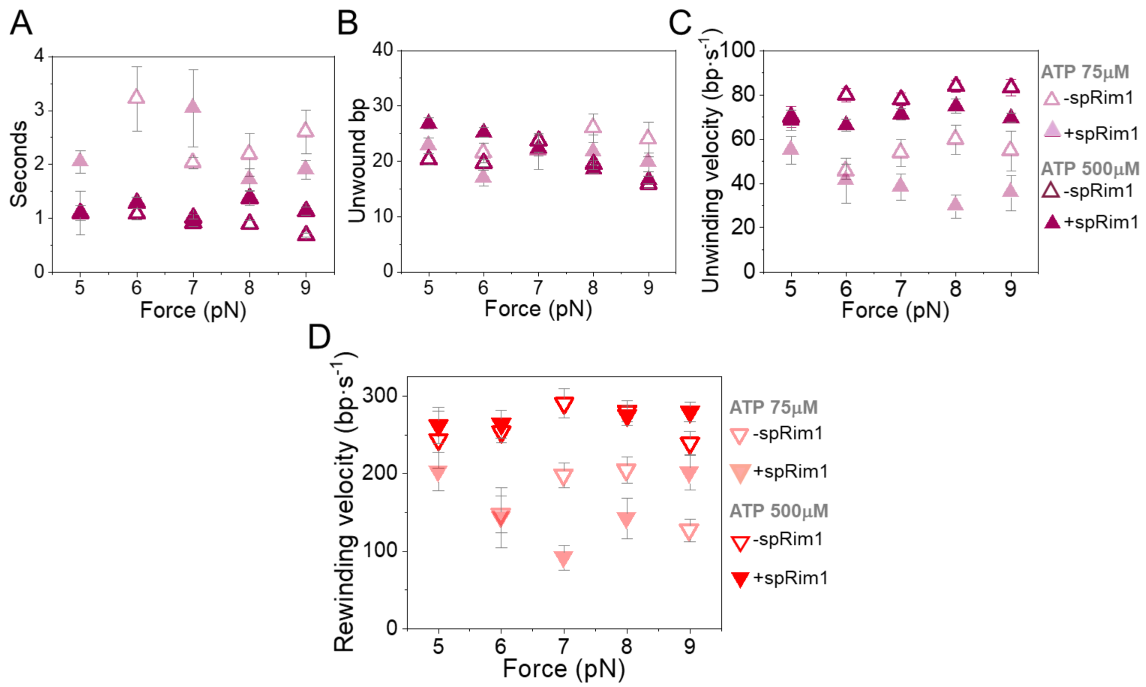

Figure S12: Unwinding and rewinding kinetics in the RNA-DNA fork: A) to **C)** Average time between unwinding bursts (A), unwinding processivity (B), and average unwinding velocity (C) as a function of applied force, measured at 75 and 500 µM ATP in the absence and presence of spRim1. **D)** Average rewinding (D) velocities as a function of force, measured at 75 and 500 µM ATP in the absence and presence of spRim1. For all plots, error bars show the s.e.m**.**

Table S1: Number of activity bursts measured at each experimental condition.

Pfh1: [ATP] / Force	5 pN	6 pN	7 pN	8 pN	9 pN	10 pN
5 µM	-	6	18	32	26	-
25 µM	20	31	49	78	73	29
50 µM	45	135	154	174	78	9
75 µM	-	12	82	117	103	-
100 µM	98	110	220	214	103	81
150 µM	-	32	106	139	80	54
250 µM	27	208	214	274	358	239
500 µM	117	154	197	241	111	51
1000 µM	-	63	93	151	159	28

Pfh1 + Rim1: [ATP] / Force	5 pN	6 pN	7 pN	8 pN	9 pN	10 pN	11 pN
25 µM	101	93	104	117	139	103	123
50 µM	-	9	8	23	-	51	27
75 µM	73	129	127	213	166	126	-
100 µM	36	57	29	15	-	-	-
150 µM	31	77	146	167	104	147	108
250 µM	107	285	414	485	312	375	179
500 µM	125	263	354	155	308	314	178
1000 µM	102	73	136	192	278	133	45

RNA-DNA, Pfh1: [ATP] / Force	5 pN	6 pN	7 pN	8 pN	9 pN	10 pN
75 µM	-	30	71	94	70	-
500 µM	37	162	119	195	114	-
**RNA-DNA, Pfh1 + Rim1: [ATP] / Force**	**5 pN**	**6 pN**	**7 pN**	**8 pN**	**9 pN**	**10 pN**
75 µM	34	13	22	27	43	63
500 µM	78	117	173	187	171	64

2 nM Alexa488(IgG)-Pfh1 ssDNA	10 pN	15 pN	20 pN	25 pN
100 µM	2	19	9	6
150 µM	2	3	6	3
250 µM	-	8	6	-
375 µM	2	8	4	2
500 µM	2	9	2	3
1000 µM	2	12	9	5
1500 µM	8	9	9	14

Table S2: Nucleic acid sequences: A) Nucleotide sequence of the unwinding segment. The 5’-terminal poly(dT)$_{30}$ serves as the helicase loading site. The strand is functionalized at the 5’ end with a biotin moiety ([Biot]) for coupling to streptavidin-coated beads in the experimental configuration shown in Figure 1B. **B)** Sequence of the first 20 ribonucleotides of the displaced strand forming the hybrid RNA-DNA fork.

A

5’[Biot]ttttttttttttttttttttttttttttttcaatcacttcaggtagcataattcatgttcattctaatctattctattgattg
aagattttactaattacaaacatctaattttaaacaaatgtttatttgattatgaatgtatcataattcggatgttca
ttctaatctattctattgattgaagattttactaattacaaacatctaattttaaacaaatgtttatttgattatgaatg
tatcataattcggcggatgttcattctaatctattctattgattgaagattttactaattacaaacatctaattttaaa
caaatgtttatttgattatgaatgtatcataattcggcggcggatgttcattctaatctattctattgattgaagatttt
actaattacaaacatctaattttaaacaaatgtttatttgattatgaatgtatcataattcggcggcggcggatgt
tcattctaatctattctattgattgaagattttactaattacaaacatctaattttaaacaaatgtttatttgattatga
atgtatcataattgtcgagccgatgcacg3’

B 3’- rGrUrUrArGrUrGrArArGrUrCrCrArUrCrGrUrArG -5’
